# Nanoscopic Imaging the Lithiation of Sulfur Nanoparticles under Electron Beam Irradiation

**DOI:** 10.1002/advs.202519640

**Published:** 2026-02-19

**Authors:** Rui Huang, Xingyu Zhang, Xingqi Liao, Ruilin Bai, Xianqi Wu, Junlei Xiang, Sheng Cheng, Yifan Zhang, Jing Wang, Hongfa Xiang, Juyeong Kim, Qiaobao Zhang, Yu Yao, Xiaohui Song

**Affiliations:** ^1^ Department of Materials Science and Engineering Hefei University of Technology Hefei Anhui P. R. China; ^2^ School of Mathematics Statistics and Mechanics Department of Engineering & Mechanics Beijing University of Technology Beijing P. R. China; ^3^ School of Materials Science and Engineering Harbin Institute of Technology (Shenzhen) Shenzhen P. R. China; ^4^ Hefei National Research Center for Physical Sciences at the Microscale Department of Materials Science and Engineering University of Science and Technology of China Hefei Anhui P. R. China; ^5^ Instrumental Analysis Center Hefei University of Technology Anhui P. R. China; ^6^ Department of Chemistry Gyeongsang National University Jinju South Korea; ^7^ Research Institute of Advanced Chemistry Gyeongsang National University Jinju South Korea; ^8^ State Key Laboratory of Physical Chemistry of Solid Surfaces College of Materials Xiamen University Xiamen P. R. China; ^9^ Engineering Research Center of High‐Performance Copper Alloy Materials and Processing Ministry of Education Hefei University of Technology Hefei P. R. China

**Keywords:** electron irradiation, In‐situ TEM, Li‐S battery, lithiation of sulphur, volume expansion

## Abstract

In situ Transmission Electron Microscopy (TEM) provides powerful insights into the reaction mechanisms of Lithium‐Sulfur (Li‐S) batteries. However, distinguishing intrinsic electrochemical behaviors from artifacts induced by high‐energy electron beam irradiation remains a critical challenge. Here, we systematically investigate the lithiation kinetics of sulfur nanoparticles triggered exclusively by electron beam irradiation, decoupling beam effects from electrochemical driving forces. We first conduct control experiments on pure lithium oxide (Li_2_O) and pure sulfur to assess their stability under electron irradiation, and then monitor lithiation behavior in a mixed system of sulfur and lithium oxide (Li_2_O), under varying irradiation times and temperatures. We report a striking “explosive” lithiation phenomenon, characterized by a massive volume expansion of up to 8300% and rapid kinetics (19312 nm^2^ s^−1^), which starkly contrasts with the ∼80% expansion observed in conventional electrochemical cycling. By conducting comparative experiments across a wide temperature range (25°C to −150°C), we identify the thermal effect of the electron beam as the dominant driving force; notably, the explosive reaction is completely suppressed at cryogenic temperatures (−150°C). Furthermore, we observe unique beam‐induced artifacts, including directional cavity formation and rapid phase transitions from crystalline S to amorphous Li_2_S. This work establishes a critical baseline for distinguishing beam‐induced damage from genuine electrochemical reactions in in situ TEM studies and provides nanoscopic insights into the thermal runaway mechanisms of sulfur cathodes under high‐energy abuse conditions, underpinning accurate characterization of Li‐S battery materials and development of advanced battery systems.

## Graphical abstract

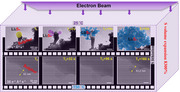

## 1 Introduction

Lithium‐sulfur batteries are promising for high‐performance energy storage due to their high capacity [1, 2, 3] and sulfur's eco‐friendly [4, 5], cost‐effective properties [6, 7, 8, 9]. However, lithiation causes volume expansion (approximately 80%) and polysulfide (Li_2_S_x_, 4 ≤ x ≤ 8) diffusion, leading to stability issues, efficiency loss, and safety risks [10, 11]. Elucidating these mechanisms is crucial for advancing Li‐S battery technology.

Despite substantial research into the lithiation process of sulfur, achieving a complete understanding of this dynamic and complex process remains challenging [12, 13, 14]. In particular, in situ characterization techniques using transmission electron microscopy (TEM) can cause unavoidable damage to sulfur materials and their lithiation products due to high‐energy electron beam irradiation [15, 16, 17]. Such irradiation can lead to structural damage of sulfur particles, abnormal reaction kinetics, and even side reactions, which compromise the accuracy and reliability of intrinsic sample characterization [18, 19, 20]. While some studies have attempted to simulate battery operation by applying a bias voltage, neglecting the effects of electron beam irradiation in experimental design may result in biased outcomes, and hinder an accurate understanding of the actual sulfur lithiation mechanism [21, 22, 23, 24]. To elucidate these nanoscale mechanisms, in situ Transmission Electron Microscopy (in situ TEM) has emerged as an indispensable tool, enabling real‐time visualization of electrochemical reactions. Pioneering studies utilizing electrical biasing or liquid cells have successfully captured intrinsic lithiation behaviors, such as collective reaction dynamics and regulated nucleation [25, 26]. Despite these advances, a fundamental challenge in TEM characterization is often overlooked: the high‐energy electron beam is not merely a passive observation tool but an active stimulus that can alter the sample's physicochemical state. Electron beam irradiation can induce radiolysis, knock‐on damage, and significant local heating, potentially triggering artificial reactions that mimic or obscure genuine electrochemical processes [27, 28]. For instance, Li‐based compounds are highly sensitive to radiolysis, generating reactive metallic lithium species that may spontaneously react with sulfur even in the absence of an external bias [29, 30]. Without a rigorous understanding of these beam‐induced artifacts, “false positive” phenomena‐ such as beam‐driven volume expansion or phase changes‐might be misinterpreted as intrinsic battery performance [31, 32].

Here, this study investigates the impact of electron beam irradiation on the sulfur lithiation process using in situ TEM. It reveals that lithiation causes an 8300% volume expansion in sulfur nanoparticles within seconds. Electron irradiation triggers lithiation, accompanied by rapid swelling at 19312 nm^2^ s^−1^. Parameters like electron beam intensity (10–35 e Å^−2^ s^−1^) and temperature (25°C, −50°C, −150°C) were adjusted, showing that the thermal effect of electron beam irradiation plays a key role in promoting lithiation. HRTEM confirms lithiation in sulfur nanoparticles and shows the interlayer spacing of {200} planes in Li_2_S. Raman and XPS further verify lithiation in sulfur and lithium oxide. The ion effect of electron beam irradiation is less significant, as Li_2_S transforms from crystalline to amorphous. In situ TEM shows that lithiation is directional. TOF‐SIMS and molecular dynamics simulations indicate that lithiation depends on irradiation time, beam intensity, and temperature. This work provides insights into nanoscale lithiation kinetics and polysulfide behavior under electron beam irradiation, offering a methodology for multi‐technique characterization in lithium‐sulfur battery research [33, 34].

## 2 Results

### 2.1 In Situ TEM Imaging of Lithium Oxide and Sulfur Nanoparticles under Electron Beam Irradiation

**FIGURE 1:**
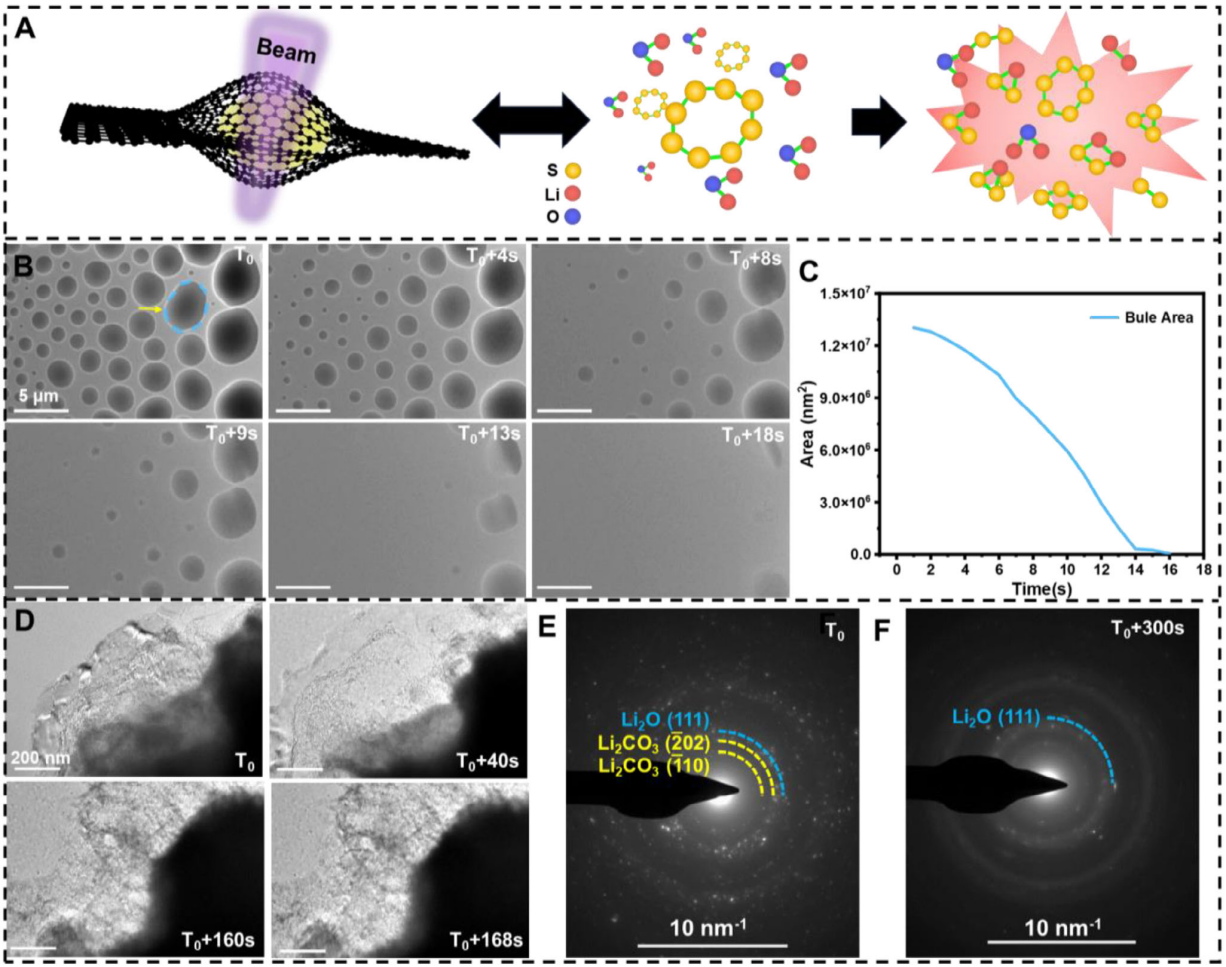
Revealing the stability of S and Li_2_O under an electron beam. (A) Schematic diagram of the reaction process of mixed lithium oxide and sulfur under TEM electron beam irradiation. (B) Snapshot image depicts the dynamic process of pure sulfur powder gradually volatilizing and disappearing from the liquid state with the extension of the TEM electron beam irradiation time. The sulfur droplets marked with the blue outline in (B) were calibrated, and the area change trend (C) was obtained. (D) The snapshot image depicts the dynamic process of lithium oxide powder gradually shrinking with the increasing duration of TEM electron beam exposure. (E) and (F) are the selected‐area electron diffraction images of lithium oxide before and after TEM electron beam irradiation. In situ TEM experimental conditions: Electron dose rate: 12.2–13.2 e Å^−2^ s^−1^; TEM model: JEM‐1400FLASH; Accelerating voltage: 120 kV; and Talos F200X, 200 kV. Temperature: 25°C.

To systematically investigate the influence of electron beam irradiation on sulfur and lithium oxide, we first examined the stability of lithium oxide and sulfur individually under electron beam irradiation, eliminating potential interference caused by their combined effects. Lithium oxide powder and sulfur powder were separately placed between graphene‐coated copper grids inside an argon‐filled glovebox to facilitate their observation under TEM. Figure 1A illustrates the reaction process of the mixed lithium oxide and sulfur under TEM electron beam irradiation. Figure 1B and Movie S1 show real‐time observations of pure sulfur under electron beam irradiation, where sulfur exhibits a liquid‐like behavior that gradually evaporates and disappears over time. The area change curve in Figure 1C was obtained by tracing the blue contour line of the sulfur droplet, revealing a contraction rate of approximately 814 000 nm^2^ s^−1^. The results indicate that sulfur nanoparticles are highly unstable under electron beam irradiation, experiencing rapid and significant volume expansion accompanied by liquidation of the solid particles [35, 36].

Similarly, pure lithium oxide (Li_2_O) powder was exposed to TEM electron beam irradiation, and a contraction phenomenon was observed, as shown in Figure 1D (Movie S2). Initially, the lithium oxide region appeared black. As the electron beam irradiation time increased, it gradually became colorless. This contraction has been attributed to the high‐energy electron beam disrupting the ionic bonds in Li_2_O, as reported in previous studies [37, 38, 39]. The interaction between electrons and the lithium (Li^+^) and oxygen (O^2−^) ions in the lattice imparts energy to these ions, leading to bond cleavage and the release of Li and O_2_ [40, 41]. Specifically, O^2−^ is oxidized to O_2_, while Li^+^ is reduced to metallic lithium. Furthermore, selected‐area electron diffraction (SAED) analysis (Figure 1E,F) revealed that the initial products, such as lithium oxide (Li_2_O) and lithium carbonate (Li_2_CO_3_), undergo a phase transformation to metallic lithium and gas under electron beam irradiation. This transformation resulted in the continuous reduction of material within the TEM observation window. On one hand, during the initial sample preparation, some lithium oxide was found to degrade into lithium carbonate, as shown in Figure S1A. On the other hand, analysis of the dynamic process, as shown in Movie S2 and corroborative data, confirmed the appearance of gas bubbles during the irradiation. These gas bubbles may originate from the reduction of Li_2_O under high‐energy electron beam irradiation, which generates oxygen gas [17].

The experimental observations indicate that the electron beam irradiation can decompose Li_2_O to produce Li. Furthermore, the longer the electron beam irradiation time, the more complete the decomposition becomes. As a result, lithium carbonate and most of the lithium oxide are decomposed, as lithium is unstable and volatile, leaving only a small amount of lithium oxide residue after 300 s of irradiation (Figure S1B).

### 2.2 In Situ TEM Observations of the Lithiation Kinetics of Sulfur Nanoparticles

**FIGURE 2:**
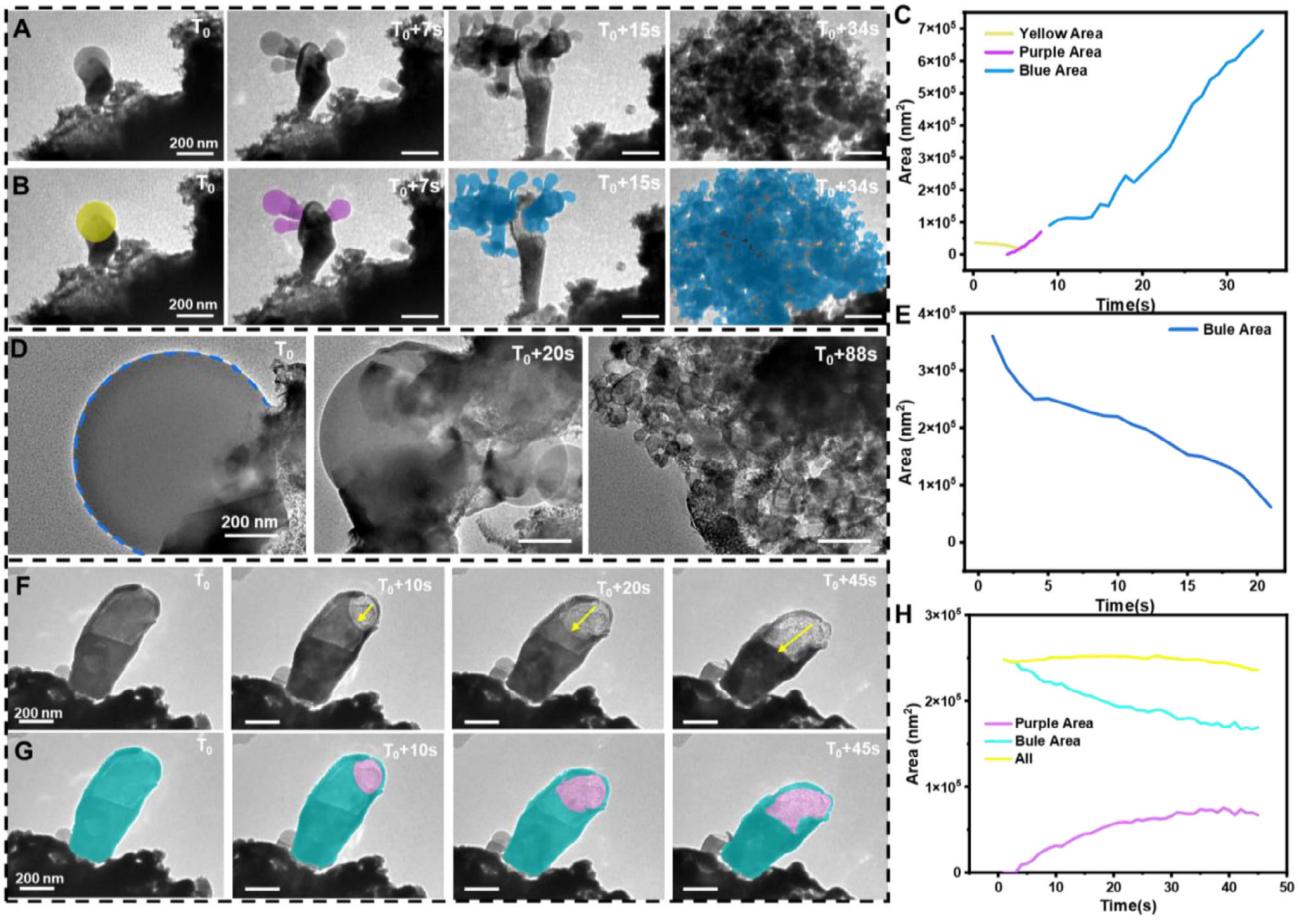
Trapping the morphology change of the S nanoparticle during lithiation. (A) The snapshot image depicts the dynamic process of lithium oxide and sulfur powder explosive lithiation with the extension of TEM electron beam exposure time; (B) The sulfur lithiation process was calibrated by color, and the area change trend was obtained. (C), yellow represents the shrinkage of the sphere at the beginning of the reaction, purple represents the initial lithiation process, and blue represents the explosive lithiation process; (D) The snapshot image depicts the dynamic process of the mixed powder of lithium oxide and sulfur shrinking and disappearing as the TEM electron beam exposure time increases, calibrate it to blue to get the area change trend (E,F) The snapshot image depicts the dynamic process of lithium oxide and sulfur powder forming directional cavities as the TEM electron beam exposure time increases;(G) The kinetic process is color‐calibrated, with purple representing the cavity and blue representing the substrate, and the area change trend (H) is obtained. In situ TEM experimental conditions: Electron dose rate: 12.2 e Å^−2^ s^−1^; TEM model: JEM‐1400FLASH; Accelerating voltage: 120 kV; Temperature: 25°C.

We systematically investigated the influence of electron beam irradiation on the lithiation process of sulfur in lithium‐sulfur batteries. Under an accelerating voltage of 120 kV, we observed the reduction of lithium oxide (Li_2_O) powder to metallic lithium (Li) under TEM electron beam irradiation. Subsequently, sulfur underwent lithiation, forming poly‐sulfur compounds (Li_2_S_x_, 2 ≤ x ≤ 8). During this reaction, sulfur exhibited significant volume expansion. While previous studies reported a sulfur expansion rate of approximately 78% [42, 43], our experiments have revealed that the volume expansion of lithiated sulfur nanoparticles reached 8300%, significantly higher than the 78% expansion typically observed in conventional sulfur cathode materials. This abnormal behavior can be attributed to three main factors: (1) the electron beam effect, where the high‐energy particle beam in TEM may promote or accelerate lithiation while causing structural changes such as lattice distortions [44]; (2) differences in experimental conditions, as TEM experiments are often conducted under high vacuum and specific temperatures, which may differ from practical battery operating environments, and may also directly observe pure sulfur nanoparticles without additives like carbon or binders [45, 46]; and (3) intrinsic properties of sulfur nanoparticles, including its high surface area and reactivity, which may lead to faster lithiation rates and more pronounced volume changes [47, 48]. As shown in Figure 2A (Figure S2 and Movie S3), sulfur underwent explosive expansion during lithiation. By analyzing the dynamic changes in surface area at different stages (Figure 2B; Figure S3), we identified three characteristic phases: initial contraction (yellow curve), early lithiation (purple curve), and explosive lithiation (blue curve). The area expansion rate was determined to be approximately 19312 nm^2^ s^−1^ (Figure 2C). Over a 34 s observation period, the area expanded nearly 19‐fold, corresponding to a volumetric swelling rate of 8300%, significantly higher than the traditional 80% expansion rate observed in sulfur electrodes during charging/discharging in lithium‐sulfur batteries. Notably, two key points should be addressed. First, the reproducibility of the in situ TEM experiments needs confirmation. In this study, we repeated the experiments and obtained consistent results, as shown in Figure S4 and Movie S4. The volume expansion rate reached 7600% in Figure S4. Second, it is important to confirm that the dynamic changes in the system are caused by lithiation rather than other factors. To verify this, we replaced lithium oxide with aluminum oxide or magnesium oxide, mixed them with sulfur powder in the same proportion, and conducted in situ TEM experiments. Unlike the Li_2_O‐S system, no significant volume expansion or explosive reaction was observed in the MgO‐S or Al_2_O_3_‐S experiments in Figure S5 and Movie S5. This confirms that the “explosive” phenomenon is driven by the lithiation reaction caused by Li_2_O decomposition.

During the experiment, two intriguing phenomena were observed. First, a large spherical particle was seen to contract (Figure 2D,E; Figure S6 and Movie S6), with a contraction rate of 14240 nm^2^ s^−1^, ultimately disappearing completely. A new sulfur particle then emerged and underwent explosive lithiation, similar to the initial process. The relationship between these two phenomena remains unclear, warranting further investigation. Second, the lithiation process was found to be directional. As shown in Figure 2F (Figure S7 and Movie S7), in a rod‐shaped sulfur particle, a cavity gradually formed and expanded along the length of the rod. Sequential imaging and analysis (Figure 2G,H; Figure S8) revealed that while the overall surface area of the rod remained relatively stable (fluctuating within ± 10000 nm^2^), the cavity area progressively increased, indicating a preferential direction of lithiation. The directional lithiation behavior observed on the products likely arises from a combination of sulfur's crystallographic anisotropy, surface energy effects, and the influence of the TEM electron beam. The above observations are consistent with theories of anisotropic surface reactions and electron‐induced surface modifications [49, 50, 51].

**FIGURE 3:**
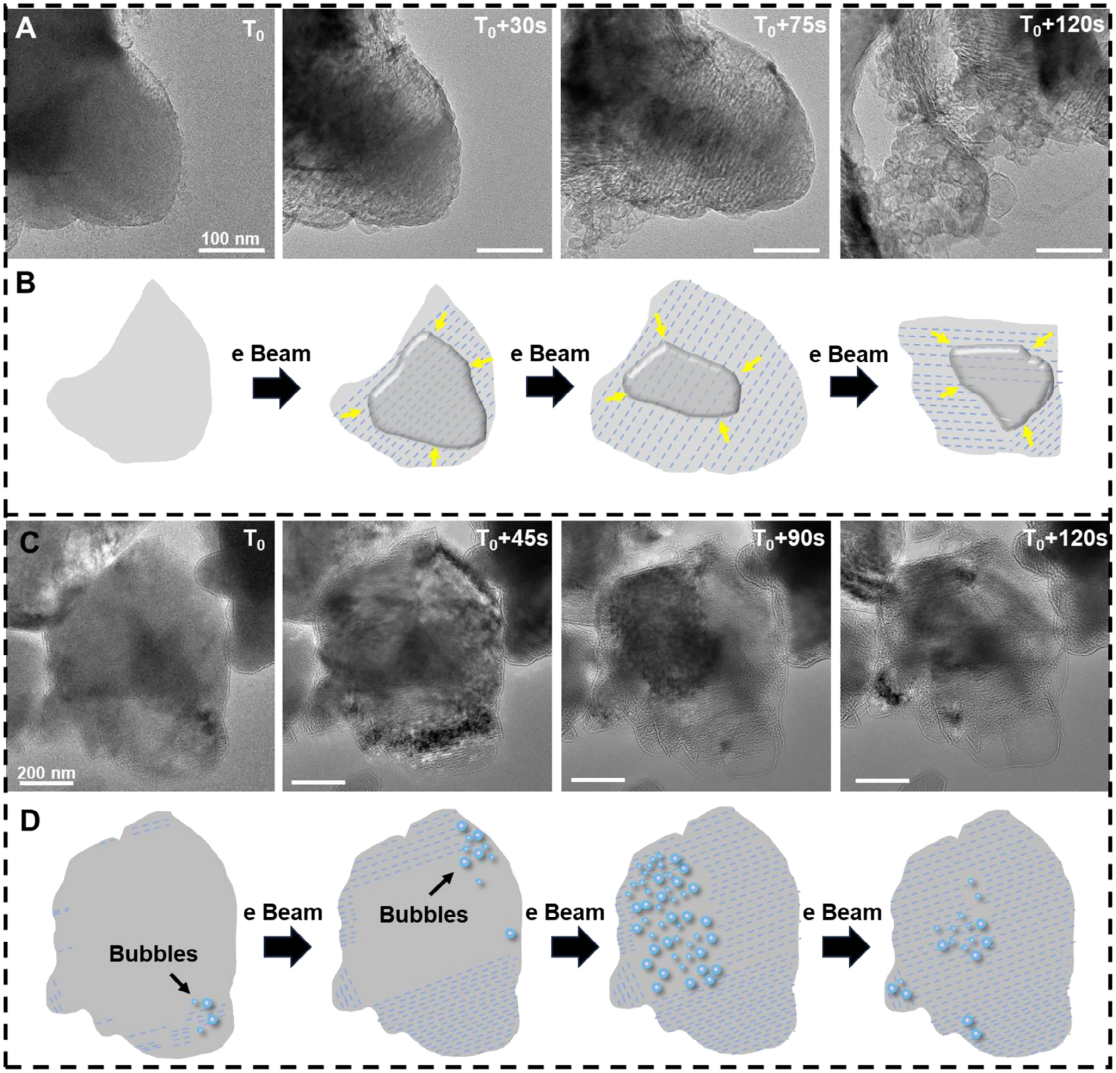
Imaging the phase change and bubbles during S nanoparticle lithiation. (A) The snapshot image depicts the dynamic process of sulfur lithiation producing a reticulated material as the HRTEM electron beam exposure time increases; (B) is a schematic representation of (A) the appearance of reticulated material during lithiation; (C) The snapshot image depicts the dynamic process of producing bubbles and reticulated matter after in sulfur lithiation as the exposure time of the HRTEM electron beam is prolonged in a different region as shown in Figure A; (D) is a schematic diagram of (C) bubbles and reticulated material during lithiation. In situ HRTEM experimental conditions: Electron dose rate: 14.5 e Å^−2^ s^−1^; TEM model: Talos F200X G2; Accelerating voltage: 200 kV; Temperature: 25°C.

At an accelerating voltage of 200 kV, distinct lithiation behavior was observed. As shown in Figure 3A (Figure S9 and Movie S8), sulfur displayed directional lithiation, proceeding from the periphery toward the center of the particle. The directed lithiation of sulfur nanoparticles under an electron beam may be attributed to the following two possible reasons: (i) Electron beam drift: Despite efforts to keep the electron beam stationary during TEM operation, it may experience slight drift [52]. If the electron beam exhibits minor, persistent directional movement (electron beam drifting), the most active reaction area (the leading edge of cavity growth) would follow the beam's movement, causing the cavities to expand in a directed manner along the beam's trajectory. (ii) Reaction‐produced gases: When gases generated by the reaction are expelled under high pressure from the cavities [53, 54], asymmetry in the exit or constraints imposed by local structures might create counterforces or directed gas flow. This could push the reaction front or soften/erode materials in a specific direction, resulting in the directed growth of cavities. Additionally, the lithiation regions left behind a network of voids with an average interlayer spacing of 6.2–6.4 nm (Figure 3B). Similar structures were observed in different regions under the same experimental conditions (Figure 3C; Figure S10 and Movie S9). The formation of these voids was attributed to the release of oxygen (O_2_) caused by the oxidation of O^2−^ under electron beam irradiation, as illustrated in Figure 3D [40, 55]. Another possible reason is that sulfur nanoparticles typically have distinct crystallographic orientations (e.g., (111) or (220) planes) [56]. The lithiation reaction may preferentially occur on specific crystal planes (or surface) due to their unique electronic and geometric properties (anisotropic surface). This can lead to the formation of directional products [57]. These findings further highlight the structural transformations and reaction mechanisms induced by electron beam irradiation during sulfur lithiation, providing valuable insights into the fundamental processes governing lithium‐sulfur battery behavior.

### 2.3 In Situ HRTEM Imaging of Phase Transformations During Sulfur Lithiation

**FIGURE 4:**
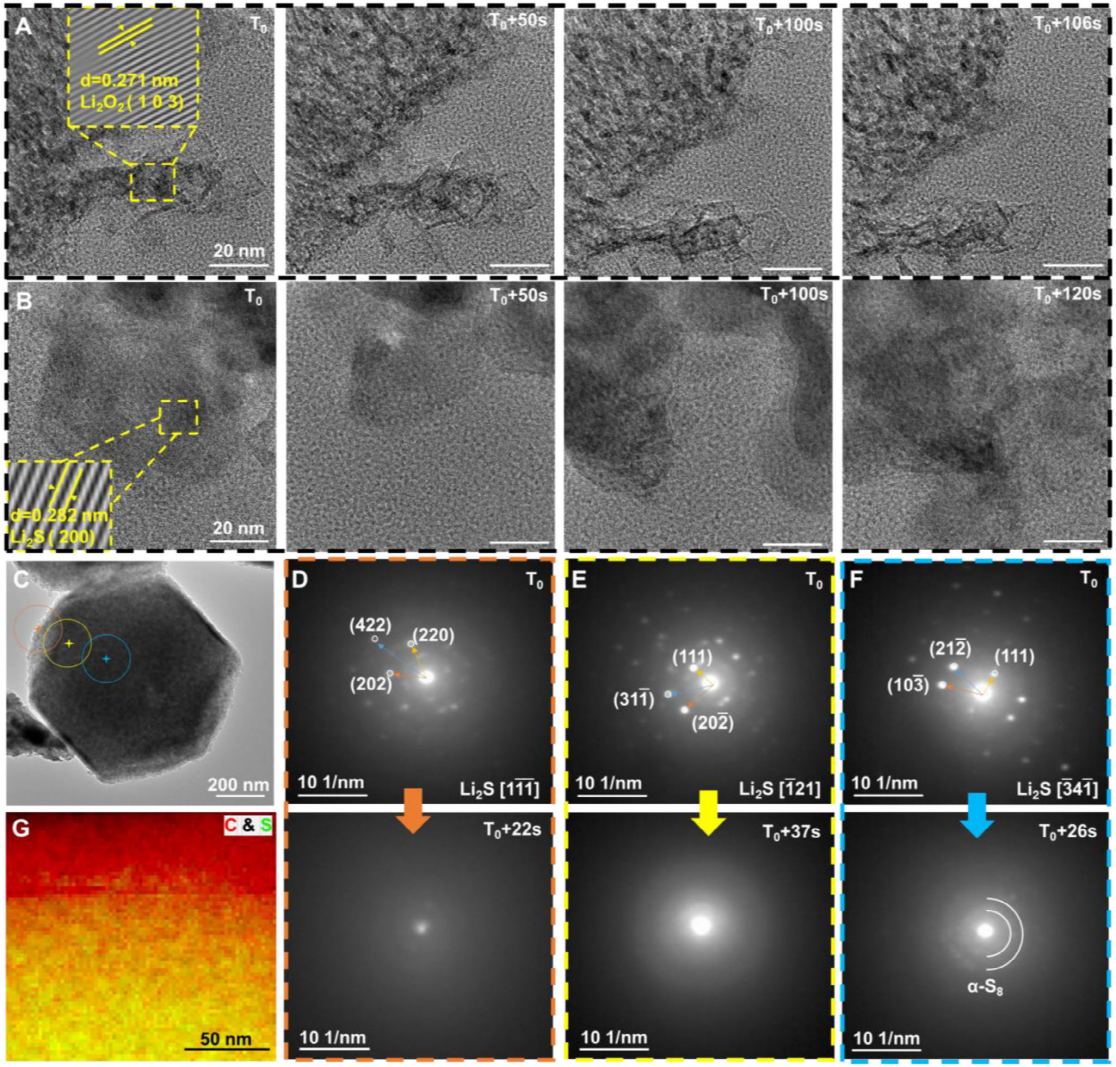
Imaging the phase transition during sulfur lithiation. (A,B) The snapshot image depicts the phase transition process resulting from sulfur lithification as the HRTEM electron beam exposure time increases, the lattice spacing in the yellow box represents Li_2_O_2_ and Li_2_S, respectively; After the capture of lithium oxide powder and S‐ZIF‐67‐C powder were mixed, the diffraction results of (D–F) were obtained by in situ nano diffraction test in different regions of the nanoparticle (C). (G) is the distribution image of C and S acquired by EELS mapping. In situ HRTEM experimental conditions: Electron dose rate: 14.5 e Å^−2^ s^−1^; TEM model: Talos F200X G2; Accelerating voltage: 200 kV; Temperature: 25°C. In situ nanodiffraction experimental conditions: Electron dose rate: 10 e Å^−2^ s^−1^; TEM model: Talos F200X G2; Accelerating voltage: 200 kV; Temperature: 25°C.

To investigate the phase transformations during sulfur lithiation, HRTEM was employed to dynamically observe the process while controlling the electron beam dose similarly. As shown in Figure 4A,B (Figures S11 and S12 and Movies S10 and S11), sulfur powder and lithium oxide powder underwent phase changes under electron beam irradiation. The magnified view of the yellow boxes in Figure 4A,B revealed the (103) plane of Li_2_O_2_ and the (200) plane of Li_2_S, respectively. This confirmed that sulfur was being lithiated into Li_2_S under TEM irradiation. To further study the behavior of sulfur within a carbon matrix, sulfur was loaded onto ZIF‐67 carbon derivative (named ZIF‐67‐C), and the mixture of Li_2_O and sulfur‐loaded ZIF‐67‐C (named S‐ZIF‐67‐C) was subjected to pseudo‐in situ nano‐diffraction tests. As shown in Figure 4C–F (Figure S13 and Movie S12–S14), the initial detection of Li_2_S diffraction spots gradually faded as the electron beam irradiation continued, with Li_2_S transforming from a crystalline to an amorphous phase. Interestingly, no S_8_ diffraction rings were observed in the outer layers of the S‐ZIF‐67‐C particles, whereas S_8_ diffraction rings were detected in the innermost regions after 30 s of electron beam shining. This indicated that sulfur in the outer layers reacted completely with Li_2_O, while an incomplete reaction occurred in the innermost layers due to reduced contact and electron beam shielding by the carbon shell. Additionally, in situ video analysis showed faster phase transformations and quicker lithiation in the outer layers, with detectable α‐S formation in the inner regions due to slower, incomplete lithiation.

After 10 min of electron beam irradiation, the EELS color map (Figure 4G) revealed the presence of sulfur signals but no detectable lithium signals. This implied that the initially formed polysulfides had decomposed, leaving only residual sulfur [3, 58]. In contrast, EELS detected weak lithium signals within the first 3 s of irradiation (Figure S14), indicating the transient presence of lithium compounds during the early stages of lithiation.

The experimental results underscore the complexity of sulfur lithiation under electron beam irradiation, with significant volume expansions, directional phase changes, and incomplete reactions depending on the spatial distribution within the carbon scaffold.

### 2.4 In Situ TEM Imaging of Lithiation Kinetics of Sulfur Loaded within ZIF‐67‐C

**FIGURE 5:**
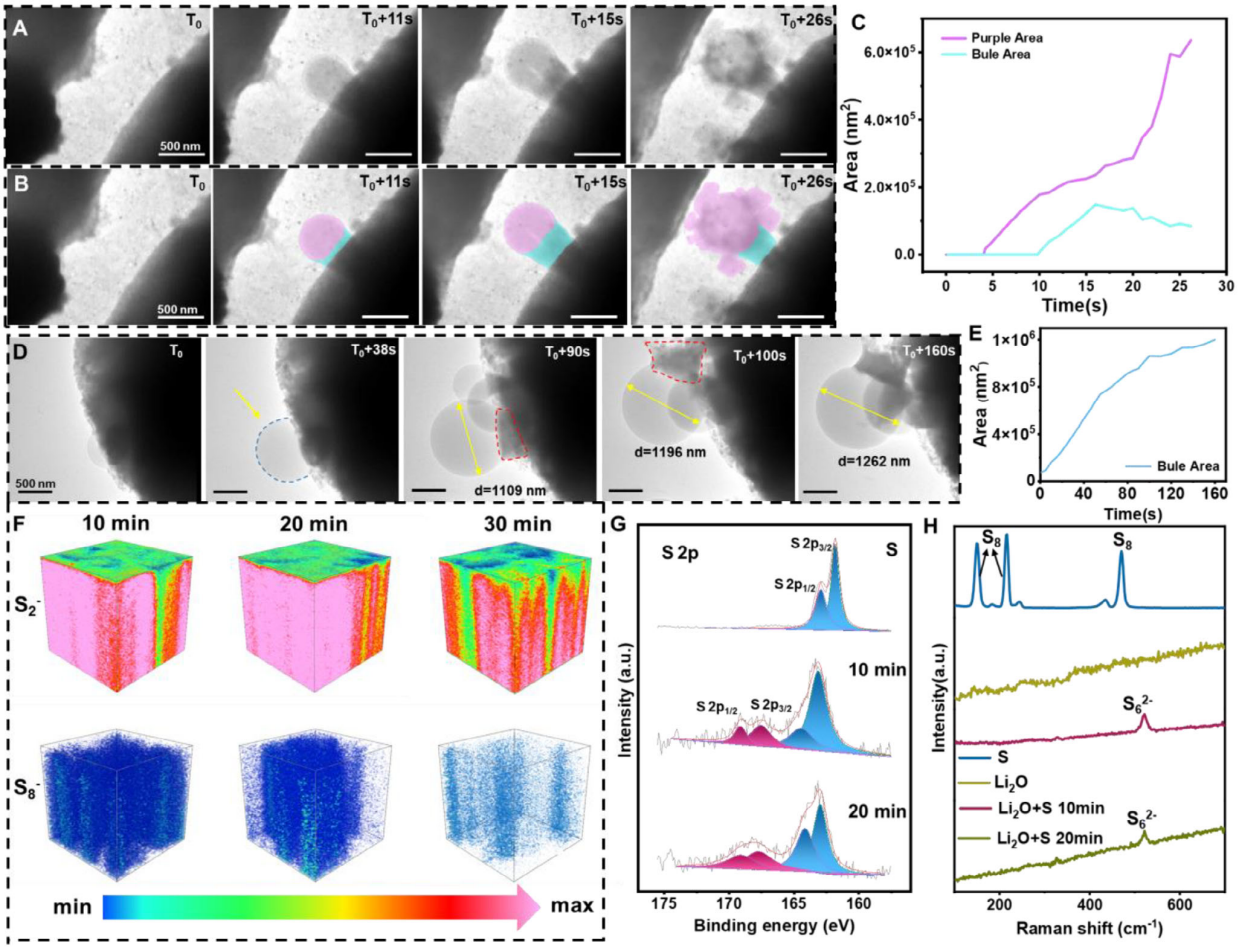
Characterizations of polysulfide lithium created by electron beam irradiation. (A) The snapshot image depicts the dynamic process of lithium oxide and S‐ZIF‐67‐C lithification as the TEM electron beam exposure time increases; (B) The dynamic process is calibrated by color, purple represents spherical material, blue represents rod‐like material, and the area change trend is obtained (C,D) The snapshot image depicts the dynamic process of lithium oxide and S‐ZIF‐67‐C spherical material breaking away from the substrate as the TEM electron beam exposure time is prolonged; The spherical substances in (D) were calibrated in blue, and the area change trend (E) was obtained. (F) TOF‐SIMS performed using TEM electron beam irradiation of 10, 20, and 30 min TEM copper mesh; (G) Images of the TEM copper mesh irradiated with pure sulfur and TEM electron beams for 10 and 20 min; (H) Raman images of TEM copper mesh irradiated with pure sulfur, pure lithium oxide, and TEM electron beams for 10 and 20 min. In situ TEM experimental conditions: Electron dose rate: 12.2 e Å^−2^ s^−1^; TEM model: JEM‐1400FLASH; Accelerating voltage: 120 kV; Temperature: 25°C.

To compare with previous studies where sulfur is loaded into porous carbon materials, we conducted in situ TEM experiments by mixing lithium oxide powder with sulfur‐loaded ZIF‐67‐C and observing its reaction kinetics under TEM. As shown in Figure 5A (Figures S15 and S17, and Movies S15 and S16), spherical and rod‐like structures gradually formed and split from the S‐ZIF‐67‐C substrate. We analyzed the area changes of the spherical (purple) and rod‐shaped (blue) materials in Figure 5B (Figures S16 and S18), obtaining the area change trend in Figure 5C, with a spherical area change rate of 23776 nm^2^ s^−1^. Unlike the direct mixing of lithium oxide and sulfur powder, the reaction in our experiment did not exhibit the explosive lithiation observed in Figure 2A. This is attributed to the reduced contact area between sulfur and lithium oxide caused by the S‐ZIF‐67‐C matrix, which slowed down the reaction rate.

Interestingly, during the in situ experiment, we observed that the spherical products continuously grew under electron beam irradiation. As shown in Figure 5D (Figure S19 and Movie S17), the spherical material remained attached to the substrate at 90 s but detached at 100 s. By measuring its area change (Figure 5E; Figure S20), we found that the area growth rate was 9862 nm^2^ s^−1^ during the growth phase while still attached to the substrate, rapidly decreasing following detachment to 2287 nm^2^ s^−1^. This significant decrease indicates that after detaching from the substrate, the lithiation reaction slowed due to the reduced supply of reactive species, leading to a decline in the growth rate.

To further understand the sulfur lithiation process, we performed TOF‐SIMS analysis on TEM copper grids irradiated for 10, 20, and 30 min (Figure 5F; Table S1). The results showed a representative depth profile of secondary ion intensity of S^2−^ and S^8−^ showing the relationship between sputter time and sputter intensity after 10, 20, and 30 min of electron beam irradiation (Figure S21). The high intensity of S^2−^ compared to the negligible signal of S^8−^ during the sputter duration indicates that elemental sulfur is effectively converted into Li_2_S_x_ within the depth range. Similarly, the decrease in internal S_8_ content suggests that sulfur within the particles also participated in the reaction. XPS and Raman analyses were conducted on samples irradiated for different durations (Figure 5G,H; Figure S22). The shift in S 2p_3/2_ and S 2p_1/2_ peaks to 163 and 164.5 eV in XPS spectra indicated the formation of Li_2_S/Li_2_S_2_, confirming sulfur lithiation under electron beam irradiation. Raman analysis revealed the disappearance of the pure sulfur (S_8_) peak and the appearance of a peak at 521 cm^−1^ attributed to S^2−^, further supporting the lithiation process [59].

Our findings demonstrate that sulfur loaded onto ZIF‐67‐C undergoes lithiation under electron beam irradiation, with the extent of lithiation increasing with irradiation time. Previous studies using TEM to investigate sulfur dynamics in lithium‐sulfur batteries under simulated battery conditions often attributed lithiation to the application of bias voltages, neglecting the potential influence of electron beam irradiation. Our work highlights the importance of considering electron beam effects in TEM‐based studies of battery dynamics, providing a valuable reference for future characterizations [60].

### 2.5 Revealing the Lithiation Kinetics of Sulfur Nanoparticle by In Situ Cryo‐TEM

**FIGURE 6:**
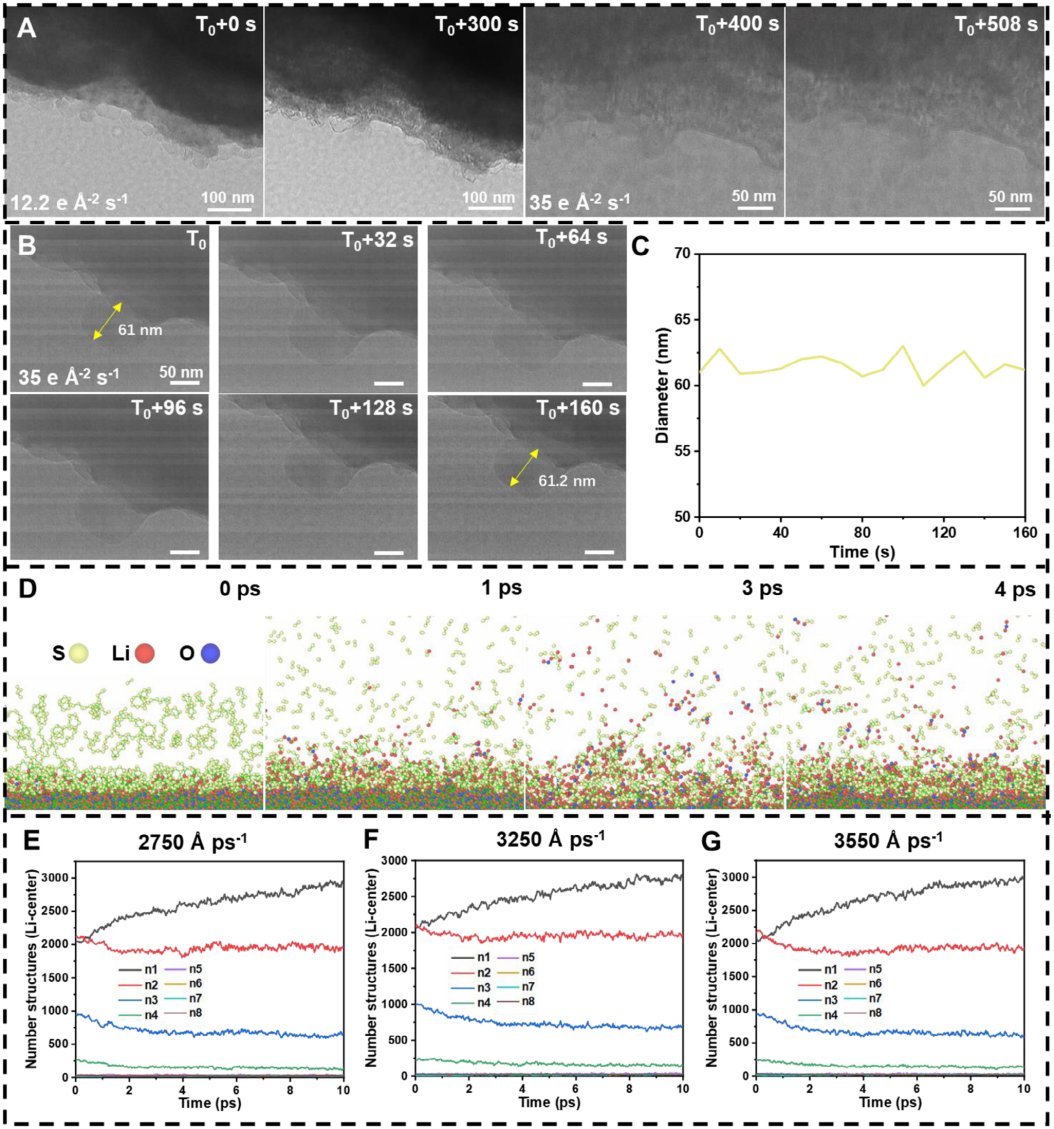
In situ freezing TEM experiments and molecular dynamics simulations of lithium oxide and sulfur. (A) The snapshot image depicts the kinetic process of a mixed powder of lithium oxide and sulfur with TEM imaging at ‐50°C; (B) The snapshot image depicts the kinetic process of a mixed powder of lithium oxide and sulfur with TEM imaging at ‐150°C; (C) Calibrate the diameter of the particles in (B) and obtain the change process; (D) Simulated kinetic images of lithium oxide and sulfur under electron irradiation; (E–G) The initial velocity is 2750, 3250 and 3550 Å ps^−1^ after colliding with the substrate, respectively, and the number of S around Li 5 Å. Cryogenic in situ TEM experimental conditions: Electron dose rate: 12.2 e Å^−2^ s^−1^, 35 e Å^−2^ s^−1^; TEM model: Talos F200C; Accelerating voltage: 200 kV; Temperature: −50°C–150°C.

To assess the impact of temperature on the lithiation kinetics of nanoscale sulfur, we conducted experiments by placing mixtures of lithium oxide and nanoscale sulfur powders into a cryogenic electron microscope operated at −50°C and −150°C. The electron dose was carefully controlled during the observations. At a dose rate of 12.2 e Å^−2^ s^−1^, no significant changes were observed in the sample (Figure 6A; Movie S18). However, upon increasing the electron beam intensity to 35 e Å^−2^ s^−1^, contraction of the lithium oxide particles was evident. This is attributed to the decomposition of lithium oxide under electron beam irradiation at −50°C, despite sulfur being in a solidified state and unable to undergo lithiation (the reaction speed is slower than that at room temperature [16, 61, 62]). The contraction of lithium oxide arises from the disruption of ionic bonds in Li_2_O by high‐energy electrons, leading to the cleavage of Li^+^ and O^2−^ ions. O^2−^ ions are reduced to O_2_, while Li^+^ ions are reduced to metallic lithium.

In contrast, at −150°C, the experimental conditions stabilized both the sulfur nanoparticles and lithium oxide particles (Figure 6B; Movie S19). No morphological changes were observed under these ultra‐cold conditions, indicating that both sulfur and lithium oxide remain stable at −150°C, effectively suppressing the electron beam‐induced effects. Further analysis revealed that the diameter of the target particles fluctuated only within ± 2 nm throughout the experiment (Figure 6C). This observation, when compared with the data obtained under conventional temperature conditions (experiments operated at room temperature), underscores the significant role of heat generated by the electron beam in promoting sulfur lithiation (Normally, the temperature is increased by 50°C–70°C under electron beam [63, 64]). The thermal effect of the electron beam is evidently dominant in this process, highlighting the importance of controlling experimental conditions to accurately interpret sulfur lithiation dynamics under electron beam irradiation.

Some of the published papers involve applying a bias voltage to simulate battery conditions and observing the lithiation process of sulfur under TEM, while others place sulfur‐carbon composites in an electrolyte containing lithium salts and use the TEM electron beam to induce the lithiation process (Table S2). Unlike previous pioneering works that utilized electrical biasing or liquid cells to visualize electrochemical lithiation, this study focuses exclusively on the lithiation kinetics triggered by electron beam irradiation in a solid‐state Li_2_O/S system (Table S3). We aim to decouple the beam‐induced effects—such as thermal runaway and explosive expansion—from intrinsic electrochemical behaviors, thereby providing a baseline for distinguishing artifacts in high‐resolution in situ characterization, which emphasizes the critical role of temperature in modulating the electron beam‐induced effects, offering valuable insights for optimizing the observation and understanding of sulfur lithiation kinetics in lithium‐sulfur battery research (Tables S4 and S5).

### 2.6 Molecular Dynamics Simulations of Sulfur Lithiation under Electron Beam Irradiation

To further investigate the mechanism of electron beam irradiation on the lithiation of nanoscale sulfur, we conducted molecular dynamics simulations to study the particle effects on sulfur lithiation. As shown in Figure 6D (Movie S20), the real‐time simulation of sulfur and lithium oxide under electron beam irradiation at room temperature demonstrated that, over time, lithium oxide and sulfur react to form various lithium‐sulfur compounds, with long‐chain sulfurs transforming into shorter chains and escaping. To explore the effects of electron beam intensity, we simulated collisions of lithium atoms with different initial velocities onto a sulfur substrate (Figure 6E–G). After 10 picoseconds of reaction, we statistically analyzed the number of sulfur atoms around lithium atoms in the vicinity of the lithium center. The results revealed that the quantities of lithium sulfides formed remained largely consistent across different particle intensities. For example, the counts of Li_x_S number ranged from 2762 to 2985, Li_x_S_2_ number ranged from 1942 to 1979, Li_x_S_3_ number ranged from 585 to 673, and Li_x_S_4_ number ranged from 125 to 148. Notably, higher‐order polysulfides such as Li_x_S_6_ and Li_x_S_8_ were found to be highly unstable and could not persist in the simulation system [65, 66]. These simulation results align well with our in situ TEM findings and other characterization results, as shown in XPS spectra and Raman spectra.

Based on these observations, we conclude that sulfur lithiation is primarily influenced by the thermodynamic effects induced by electron beam irradiation, particularly changes in room temperature, rather than electron beam‐induced effective collision. This conclusion is supported by both the in situ cryo‐TEM experiments and theoretical calculations. The consistency between experimental and computational findings underscores the dominant role of thermal effects in sulfur lithiation.

## 3 Conclusion

In summary, this study systematically decouples the effects of high‐energy electron beam irradiation from intrinsic electrochemical processes during the lithiation of sulfur nanoparticles. Our findings reveal a striking “explosive” lithiation phenomenon characterized by a massive volume expansion of up to 8300% and rapid kinetics of 19312 nm^2^ s^−1^, which stands in sharp contrast to the ∼80% expansion typically observed in conventional Li‐S battery cycling. Through a combination of in situ cryo‐TEM and molecular dynamics simulations, we identify the thermal effect of the electron beam as the dominant driving force for these rapid transformations, while the reaction is completely suppressed at cryogenic temperatures (−150°C). Furthermore, we identified unique beam‐induced artifacts, including directional cavity formation and the rapid transition of Li_2_S from a crystalline to an amorphous phase, which could be misinterpreted as genuine electrochemical behavior in standard in situ studies. By establishing this critical baseline, our work highlights the necessity of accounting for electron‐matter interactions to ensure the accuracy of nanoscale battery characterization. These insights not only provide a fundamental understanding of sulfur's thermal runaway mechanisms under high‐energy abuse but also offer a methodological framework for developing more reliable in situ techniques for next‐generation energy storage systems, potentially providing the theoretical and experimental foundations needed to drive the practical application of high‐performance lithium‐sulfur batteries [67, 68, 69, 70].

## 4 Experimental Section

### 4.1 ZIF‐67 Preparation

0.6 g of cobalt acetate tetrahydrate was dissolved in 5 mL of deionized water and sonicated for 2 min until the solid completely dissolved to obtain solution A. Subsequently, 2.24 g of 2‐methylimidazole was dissolved in 5 mL of deionized water and sonicated for 2 min until the solid fully dissolved to obtain solution B. Solution A was then poured into solution B, rapidly stirred for 20 s at room temperature, and left to stand for 24 h. Afterward, the supernatant was removed using a pipette, and the solid was washed with ethanol and deionized water by centrifugation (8000 rpm min^−1^ by three times). Finally, the solid was dried in a 55°C oven for 8 h to obtain a purple powder.

### 4.2 The Synthesis of ZIF‐67‐C

In the process of preparing N‐doped composite porous carbon materials using ZIF‐67 as a precursor through high‐temperature sintering, an argon atmosphere was chosen for sintering. ZIF‐67 weighing 500 mg was sintered at temperatures of 800°C. The heating rate was uniformly set at 5°C min^−1^, and the dwell time was uniformly set at 2 h.

### 4.3 The Synthesis of S‐ZIF‐67‐C

The carbon‐sulfur mixture with a mass ratio of 2:3 was added to a sealed reaction vessel and heated in a tube furnace at a rate of 5°C min^−1^ until reaching 155°C. It was maintained at this temperature for 12 h under an argon atmosphere. After sintering, the furnace was allowed to cool to room temperature.

### 4.4 In Situ TEM Experiments

Lithium oxide powder and sublimed sulfur powder (or S‐ZIF‐67‐C) were mixed in a 1:1 mass ratio in an argon‐filled glovebox. The mixed powder was dispersed onto TEM copper grids, and excess powder was removed using a bulb syringe. A new copper grid was placed on top, and the two grids were mounted on a TEM sample holder. The setup was then placed in a TEM (JEM‐1400FLASH, Talos F200X G2) operating at an accelerating voltage of 120 or 200 kV.

### 4.5 In Situ Cryogenic TEM Experiments

At room temperature, the mixture of lithium oxide and sulfur was diluted onto a Lacey support grid (Lacey Formvar/Carbon, 200 mesh, Cu; Ted Pella, Inc.). The samples were prepared in a custom‐built environmental chamber under controlled temperature and humidity conditions (97%–99%). The support grids were dipped into liquid ethane, which was cryogenically cooled with liquid nitrogen. The grids were transferred using a freeze‐transfer device onto a Gatan 626 cryo‐holder and loaded into the Talos F200C TEM instrument (200 kV). Imaging was conducted using an SC 1000 CCD camera (Gatan, Inc., USA) at temperatures ranging from −50°C to−150°C under an accelerating voltage of 200 kV. Electron beam intensity was maintained at 120–200 kV with a dose rate of 12.2–35 (e Å^−2^ s^−1^). Data analysis was carried out using Digital Micrograph software.

### 4.6 TOF‐SIMS Characterization

S‐ZIF‐67‐C was mixed with a 10% PVDF solution, coated onto TEM copper grids, and dried in a 55°C oven for 6 h before being transferred to a glovebox. Lithium oxide powder was then dispersed onto a clean copper grid, which was placed over the S‐ZIF‐67‐C‐coated grid. The two copper grids were mounted onto a TEM sample holder and exposed to the electron beam for 10, 20, and 30 min, respectively. After exposure, the upper copper grid containing lithium oxide was removed, and TOF‐SIMS analysis was performed on the lower grid coated with powder.

### 4.7 XPS and Raman Characterization

The lithium oxide powder and sulfur powder are thoroughly mixed in a glovebox and then dispersed between two TEM copper grids. The samples are exposed to the TEM electron beam for 10, 20, and 30 min, respectively. After the exposure, the top copper grids are removed, and the bottom one is placed on the XPS and Raman sample stages for testing.

### 4.8 Molecular Dynamics Simulation

The molecular dynamics simulation was conducted using the open‐source LAMMPS software [71]. The simulation box dimensions were set to 10.2 × 8.3 × 81.7 nm (x‐y‐z directions), containing a total of 118525 atoms, including 923 S_8_ rings randomly distributed between two layers of lithium atoms. The simulation employed the ReaxFF reactive force field, with parameters derived from [72]. The irradiation damage simulation timestep was set to 0.0001 ps, and periodic boundary conditions were applied in all three directions. Before the collision, the system was relaxed using the canonical (NVT) ensemble for 5 ps. Five lithium atoms were randomly selected as primary knock‐on atoms (PKA) in the center of the box and were projected toward the substrate with initial velocities of 2750, 3250, and 3550 Å ps^−1^. The collision process was performed under the microcanonical ensemble (NVE), and to closely observe the collision dynamics, the structure files were saved every 0.02 ps. Visualization and analysis of the simulation were carried out using Ovito software [73].

## Author Contributions

Prof. Xiaohui Song (X.S) conceived the idea and directed the project. R.H., X.Z., X.L., and X.S. designed all the experiments and synthesis of the carbon materials. X.S., R.H., Y.Z., X.L., D.Y., and C.S. designed the in situ TEM experiments and the data collection. X.S., R.H., J.W., X.Z., X.W., J.K., and J.X., performed the imaging process. X.S., H.X., Q.Z., R.B., and Y.Y., performed and analyzed Time of Flight Secondary Ion Mass Spectrometry (TOF‐SIMS). X.S., Y.Z., J.W., and X.Z. discussed and conducted MD simulation followed by data analysis. All in situ liquid phase TEM experiments and data collections are conducted at Hefei University of Technology, China. The manuscript preparation is completed at Hefei University of Technology. All authors commented on the manuscript and contributed to the discussion of the results, the preparation, and revision of the manuscript.

## Conflicts of Interest

The authors declare no competing interests.

## Supporting information




**Supporting File 1**: advs74475‐sup‐0001‐MovieS1.mp4;

**Supporting File 2**: advs74475‐sup‐0002‐MovieS2.mp4;

**Supporting File 3**: advs74475‐sup‐0003‐MovieS3.mp4;

**Supporting File 4**: advs74475‐sup‐0004‐MovieS4.mp4;

**Supporting File 5**: advs74475‐sup‐0005‐MovieS5.mp4;

**Supporting File 6**: advs74475‐sup‐0006‐MovieS6.mp4;

**Supporting File 7**: advs74475‐sup‐0007‐MovieS7.mp4;

**Supporting File 8**: advs74475‐sup‐0008‐MovieS8.mp4;

**Supporting File 9**: advs74475‐sup‐0009‐MovieS9.mp4;

**Supporting File 10**: advs74475‐sup‐0010‐MovieS10.mp4;

**Supporting File 11**: advs74475‐sup‐0011‐MovieS11.mp4;

**Supporting File 12**: advs74475‐sup‐0012‐MovieS12.mp4;

**Supporting File 13**: advs74475‐sup‐0013‐MovieS13.mp4;

**Supporting File 14**: advs74475‐sup‐00014‐MovieS14.mp4;

**Supporting File 15**: advs74475‐sup‐00015‐MovieS15.mp4;

**Supporting File 16**: advs74475‐sup‐00016‐MovieS16.mp4;

**Supporting File 17**: advs74475‐sup‐00017‐MovieS17.mp4;

**Supporting File 18**: advs74475‐sup‐00018‐MovieS18.mp4;

**Supporting File 19**: advs74475‐sup‐00019‐MovieS19.mp4;

**Supporting File 20**: advs74475‐sup‐00020‐MovieS20.mp4;

**Supporting File 21**: advs74475‐sup‐0021‐SuppMat.docx.

## Data Availability

This study did not generate new, unique reagents. The full list of chemicals and materials could be found in the supplementary information. The data that support the plots within this study are available in supplementary information or from the corresponding author (Prof. Xiaohui Song: xiaohuisong@hfut.edu.cn) upon reasonable request. Source Data are provided with this paper.
