# Supplementary material for: Nanoscopic Imaging the Lithiation of Sulfur Nanoparticles under Electron Beam Irradiation
Source: Adv Sci (Weinh). 2026 Feb 19;13(24):e19640. doi: 10.1002/advs.202519640 (PMC13116160; doi:10.1002/advs.202519640)
Supplement: Supplementary file 21 — Supporting File 21: advs74475‐sup‐0021‐SuppMat.docx. [file ADVS-13-e19640-s021.docx]

***Supplementary Information***

***for***

**Nanoscopic Imaging the Lithiation of Sulfur Nanoparticles under** **Electron Beam Irradiatio*n***

Rui Huang^1,†^, Xingyu Zhang^2,†^, Xingqi Liao^3,†^, Ruilin Bai^4^, Xianqi Wu^1^, Junlei Xiang^1^, Sheng Cheng^5^, Yifan Zhang^1^, Jing Wang^1^, Hongfa Xiang^1^, Juyeong Kim^6,7^, Qiaobao Zhang^8^, Yu Yao^4^, Xiaohui Song^1,9,*^

^1^Department of Materials Science and Engineering, Hefei University of Technology, Hefei, Anhui Province 230009, China

^2^School of Mathematics, Statistics and Mechanics, Department of Engineering & Mechanics, Beijing University of Technology, Beijing, 100124, China

^3^School of Materials Science and Engineering, Harbin Institute of Technology (Shenzhen), Shenzhen, 518055, China

^4^Hefei National Research Center for Physical Sciences at the Microscale, Department of Materials Science and Engineering, University of Science and Technology of China, Hefei, Anhui, 230026, China

^5^Instrumental Analysis Center, Hefei University of Technology, Anhui Province, 230009, P.R. China.

^6^Department of Chemistry, Gyeongsang National University, Jinju 52828, South Korea

^7^Research Institute of Advanced Chemistry, Gyeongsang National University, Jinju 52828, South Korea

^8^State Key Laboratory of Physical Chemistry of Solid Surfaces College of Materials Xiamen University Xiamen 361005 China

^9^Engineering Research Center of High-Performance Copper Alloy Materials and Processing, Ministry of Education, Hefei University of Technology, Hefei 230009, China

^†^These authors contribute equally to this work

*Address correspondence to: [xiaohuisong@hfut.edu.cn](mailto:xiaohuisong@hfut.edu.cn)

**This file includes:**

**1**. Experimental and Methods

**2**. Figures S1–S22

**3**. Tables S1–S5

**4**. Movies S1–S20

**5**. References

**1. Experimental and Methods**

**Chemicals.** All chemical reagents were used as purchased without further purification. Cobalt acetate tetrahydrate ((CH_3_COO)_2_Co·4H_2_O, Analytical Pure (AR)); Sulfuric acid (H_2_SO_4_, Analytical Pure (AR));ethanol (EtOH, Analytical Pure (AR)) all from China National Pharmaceutical Group Corporation; 2-methylimidazole (C_4_H_6_N_2_, Alfa Aesar, 98%);Sublimed sulfur (S_8_, Aladdin，99.95%);Lithium oxide (Li_2_O, Macklin, 99.9%).

**In Situ TEM Experiment of Pure Nanosulfur/Pure Lithium Oxide Mixtures.**

To investigate the interaction between sulfur and lithium oxide under in situ TEM observations, the following procedure was conducted:

1. Sample Preparation:

Dispersing powders: Sulfur powder and lithium oxide powder were carefully dispersed onto TEM copper grids using a pipette to ensure even distribution on the grid surface.

Removing excess powder: Any excess powder that did not adhere to the grid was gently removed using a bulb pipette, ensuring minimal disturbance to the sample.

Covering with a new grid: A second TEM copper grid was placed on top of the sample, creating a sandwich-like structure to protect the sample during transfer and examination.

1. TEM Analysis:

Sample placement and observation setup: The prepared sample, consisting of the double-layered copper grids, was securely positioned onto a TEM sample holder.

Instrument setup: The sample was inserted into a JEM-1400FLASH TEM operating at an accelerating voltage of 120 kV. The magnification was set between 40000 - 60000 times to capture the dynamic interaction between sulfur and lithium oxide at the nanoscale.

1. Recording the Kinetic Process:

Throughout the experiment, the kinetic changes occurring between the sulfur and lithium oxide were continuously monitored and recorded by capturing high-resolution images and videos. This allowed for the detailed observation of any structural changes, reactions, or morphological transformations in real-time, under controlled conditions within the TEM.

**Analysis of TEM movies via deep learning.** TEM (Transmission Electron Microscopy) movies processing analysis using Dragonfly deep learning involves employing advanced artificial intelligence techniques to extract valuable insights and information from dynamic sequences of TEM images, often referred to as "movies." Dragonfly, a deep learning framework, enhances the analysis of these movies by automating various tasks and Key Steps in TEM Movie Processing Analysis with Dragonfly Deep Learning in the following procedures as we developed before^1^: (1) Data Preparation: The first step involves preparing the TEM movie data for analysis. This includes converting the movie frames into a suitable format, ensuring proper alignment, and potentially applying pre-processing steps such as noise reduction or contrast enhancement. (2) Training Deep Learning Models: Dragonfly deep learning involves training neural network models to recognize and classify specific features or objects of interest within the TEM movie frames. This training process requires annotated data where these features are manually labeled. For example, if the goal is to track nanoparticles or structural changes in the movie, the neural network is trained to identify and locate them accurately. (3) Feature Extraction. (4) Object Tracking. (5) Segmentation and Masking. (6) Quantitative Analysis: dragonfly can perform quantitative measurements on the extracted features, such as particle size distribution, velocity, intensity changes, and more. These measurements provide valuable insights into the underlying phenomena being studied.


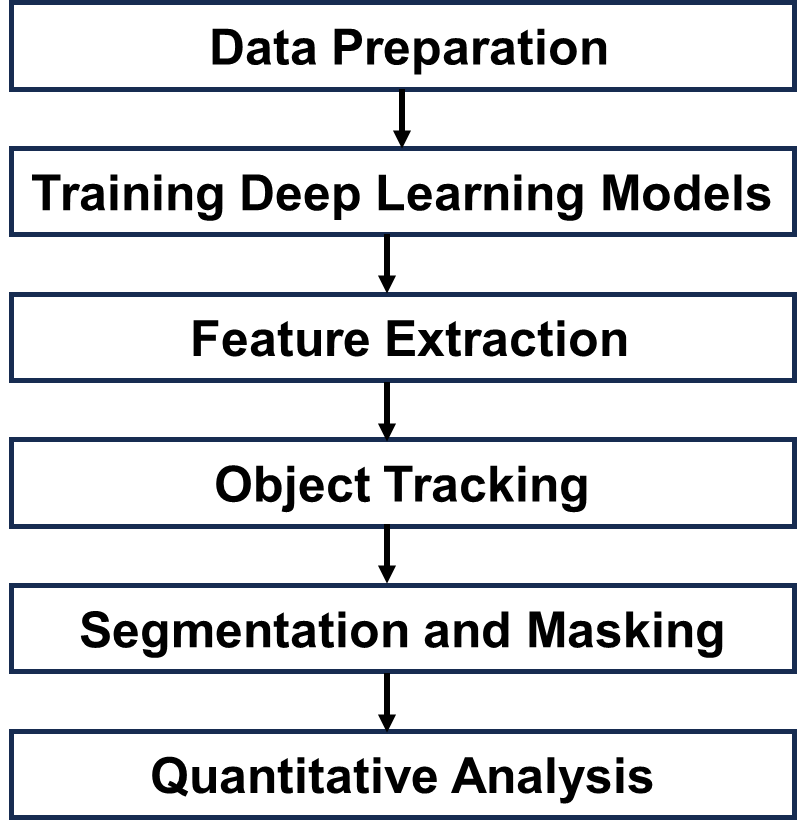


**2. Supplementary Figures S1-22**

**
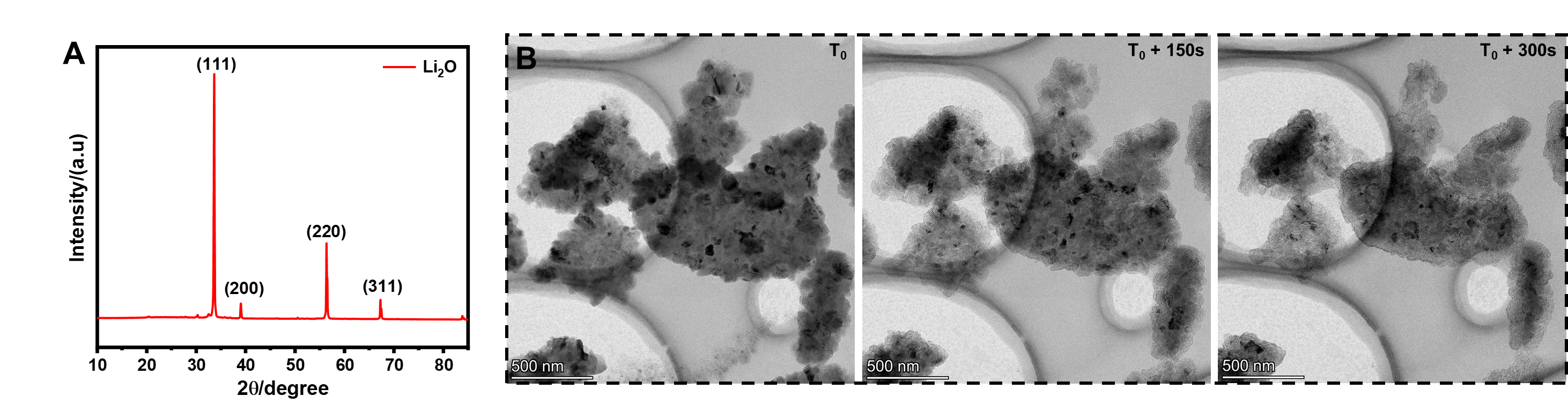
**

**Figure S1. Revealing the stability of Li_2_O under electron beam**. **(A)** XRD spectra of lithium oxide powder, showing the presence of a small amount of lithium carbonate; **(B)** the corresponding in-situ TEM BF images (as shown in **Figures 1E** and **1F**) exhibit a continuous decrease in the amount of solid material.


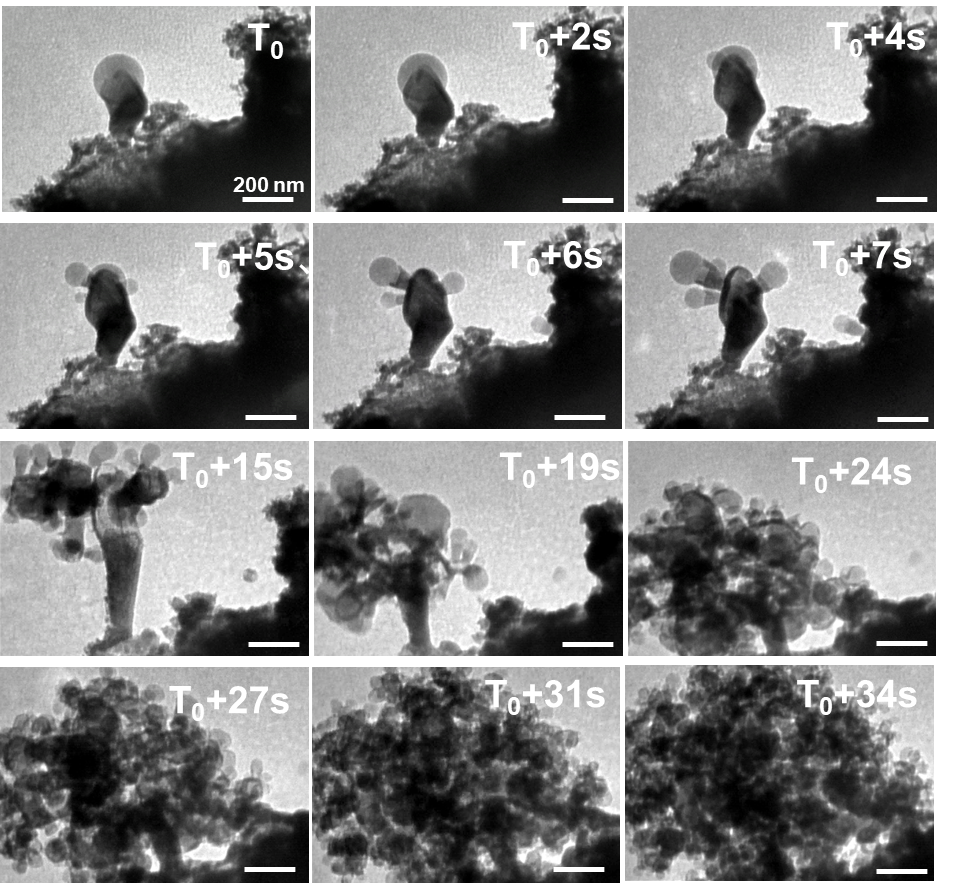


**Figure S2. Trapping the morphology change of S nanoparticle during lithiation.** The snapshot image depicts the dynamic process of lithium oxide and sulfur powder explosive lithiation with the extension of TEM electron beam exposure time, consistent with the sample in **Figure 2A**.


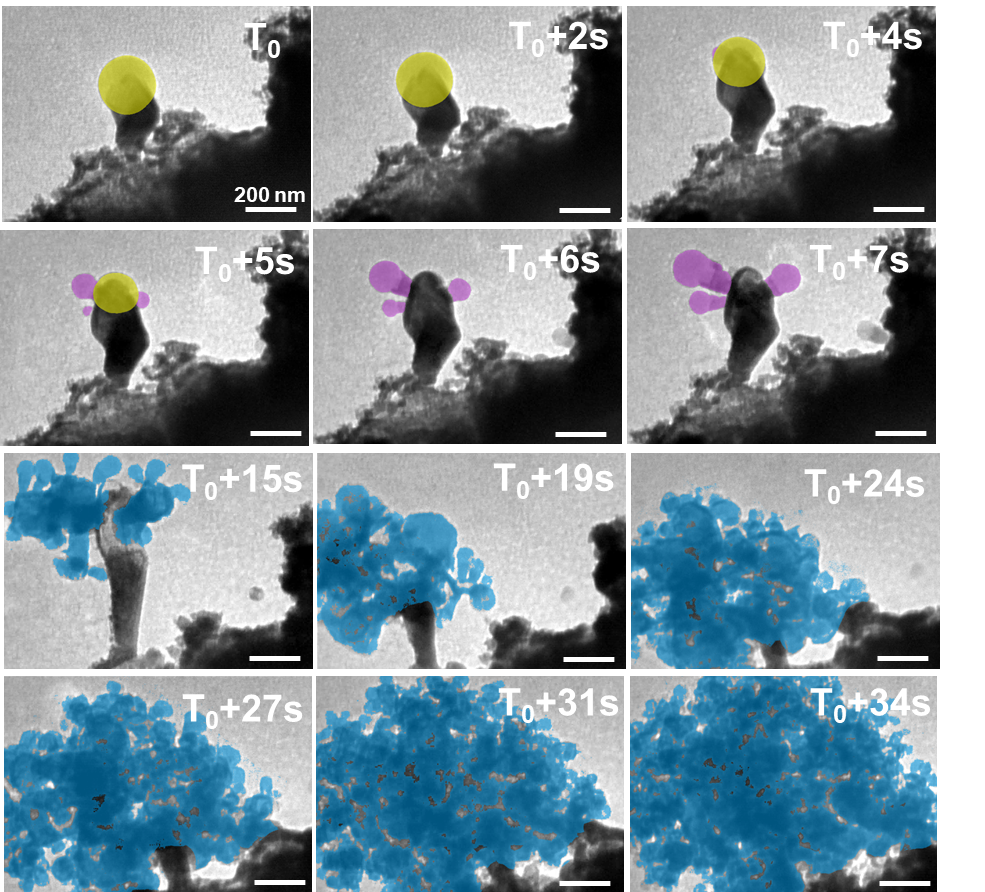


**Figure S3. Trapping the morphology change of S nanoparticle during lithiation.** The real-time images of the sulfur lithiation kinetics under TEM after mixing the lithium oxide powder and sulfur powder were color-calibrated, with yellow representing the initial shrinkage of the spheroids, purple representing the initial lithiation process, and blue representing the explosive lithiation process, consistent with the sample in **Figure 2B**.


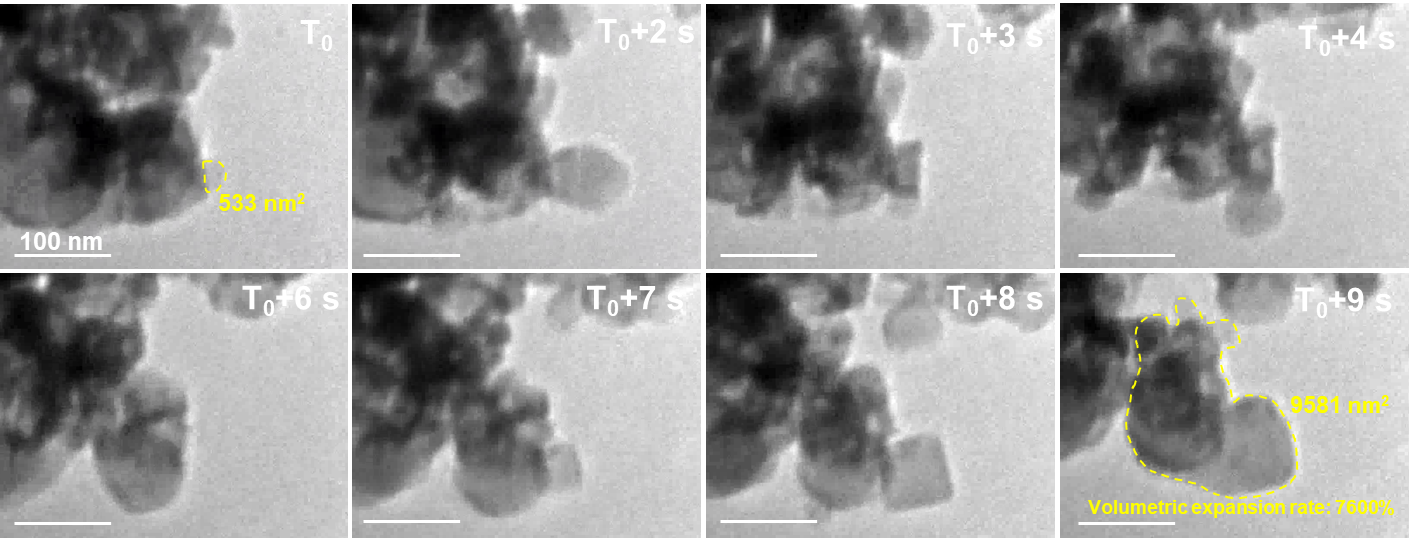


**Figure S4. Additional observation of the explosive lithiation of sulfur nanoparticles.** The time-lapse TEM snapshots capture the rapid expansion kinetics of another representative sulfur particle under electron beam irradiation (12.2 e Å^-2^ s^-1^), serving as a replicate experiment to validate the findings in **Figure 2**. The particle undergoes a dramatic morphological evolution within 9 seconds. The projected area expands from an initial 533 nm² (T_0_) to 9581 nm² (T_0_+9 s). Based on the isotropic expansion assumption, the corresponding volumetric expansion is calculated to be approximately 7600%. This result is highly consistent with the 8300% expansion reported in the main text, confirming that the beam-induced massive expansion is a robust and reproducible phenomenon.


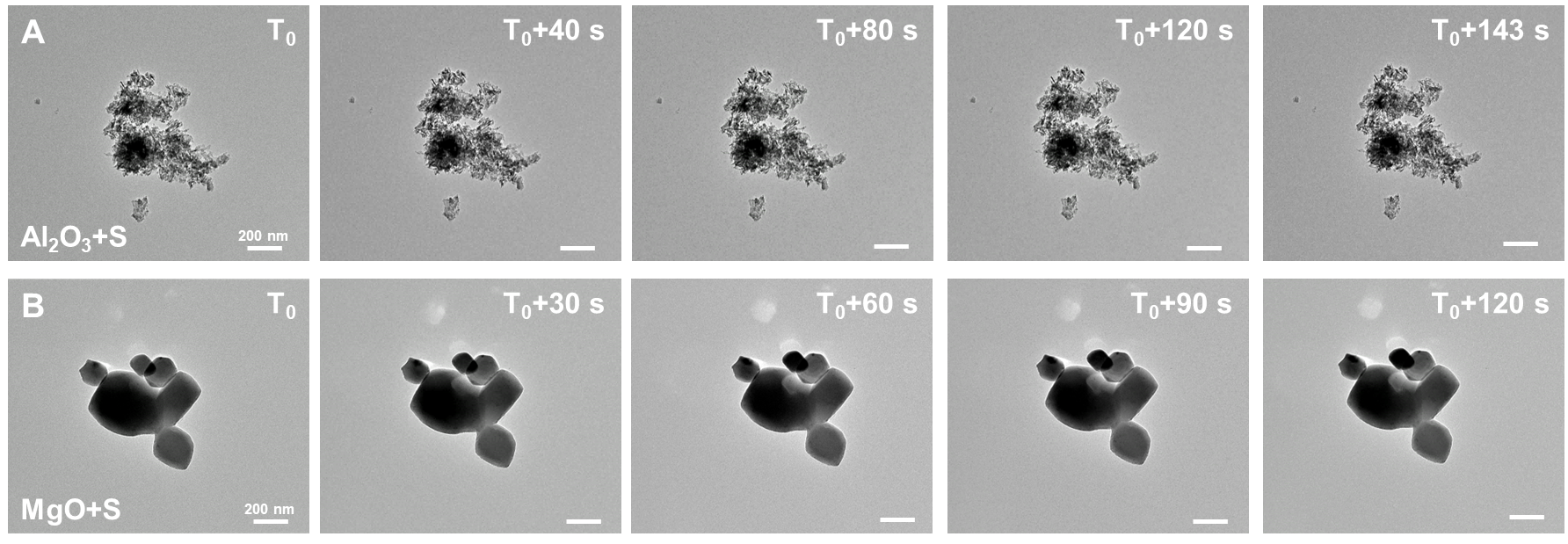


**Figure S5. Control experiments verifying the lithium-dependent nature of the explosive expansion.** Time-lapse TEM snapshots comparing the dynamic behavior of non-lithium oxide mixtures under electron beam irradiation, serving as control groups for the Li_2_O-S system. (A) The dynamic process of aluminum oxide (Al_2_O_3_) and sulfur mixed powder. No significant volume expansion or morphological change was observed over 143 seconds. (B) The dynamic process of magnesium oxide (MgO) and sulfur mixed powder under identical conditions, maintaining a stable morphology over 120 seconds.


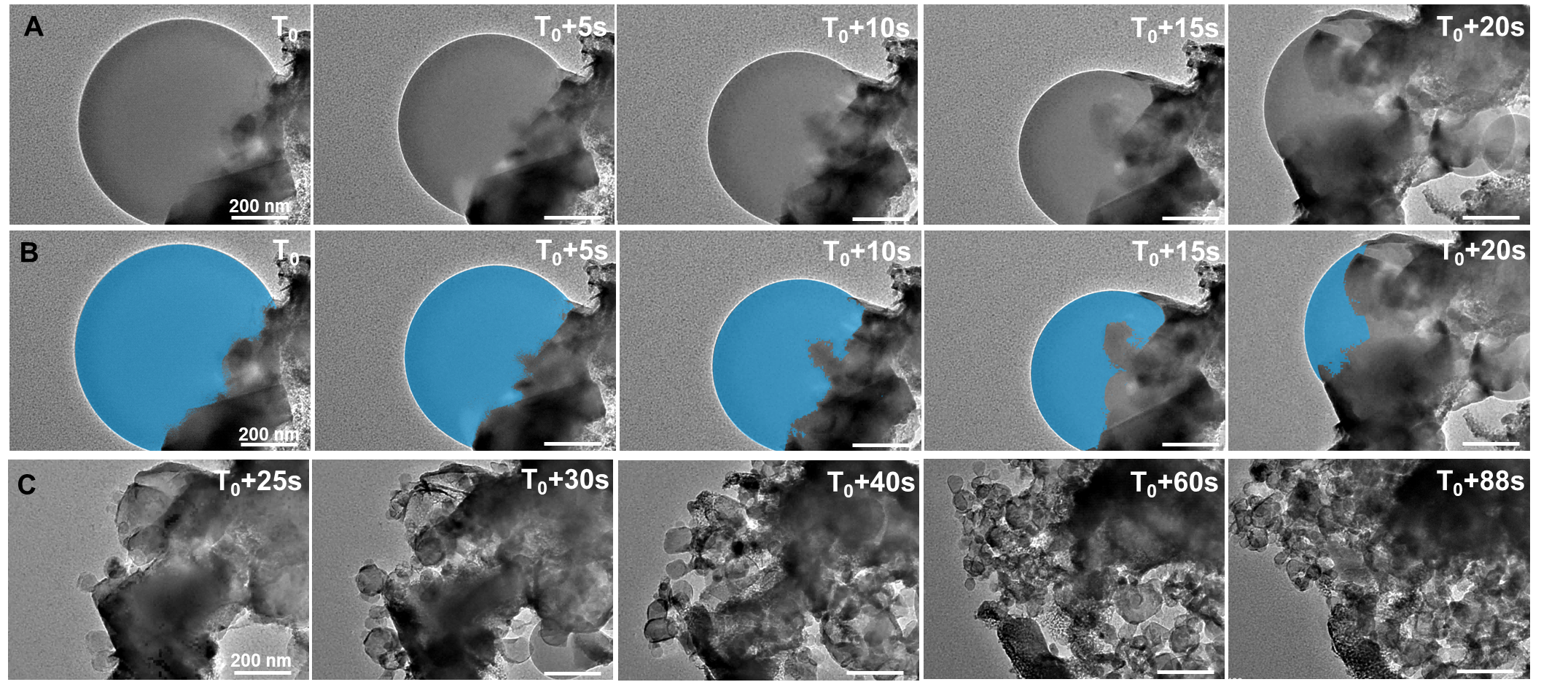


**Figure S6. Trapping the morphology change of S nanoparticle during lithiation.** **(A)** The snapshot image depicts the dynamic process of the mixed powder of lithium oxide and sulfur shrinking and disappearing as the TEM electron beam exposure time increases, consistent with the samples in **Figure 2D**; **(B)** The real-time image of the dynamics of the spheroidal material shrinking and disappearing under TEM after mixing lithium oxide powder and sulfur powder was captured, and the area change trend was obtained by calibrating it as blue; **(C)** is the subsequent lithiation change of the sample **(A)**.


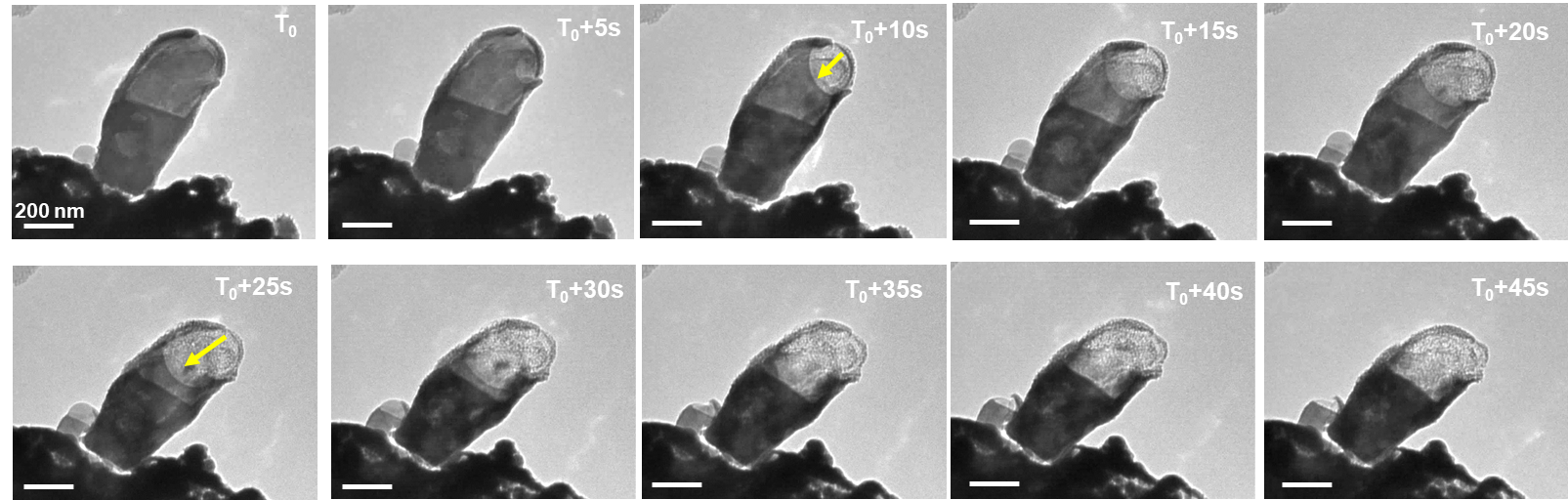


**Figure S7. Trapping the morphology change of S nanoparticle during lithiation.** The snapshot image depicts the dynamic process of lithium oxide and sulfur powder forming directional cavities as the TEM electron beam exposure time increases, Consistent with the sample in **Figure 2F**.


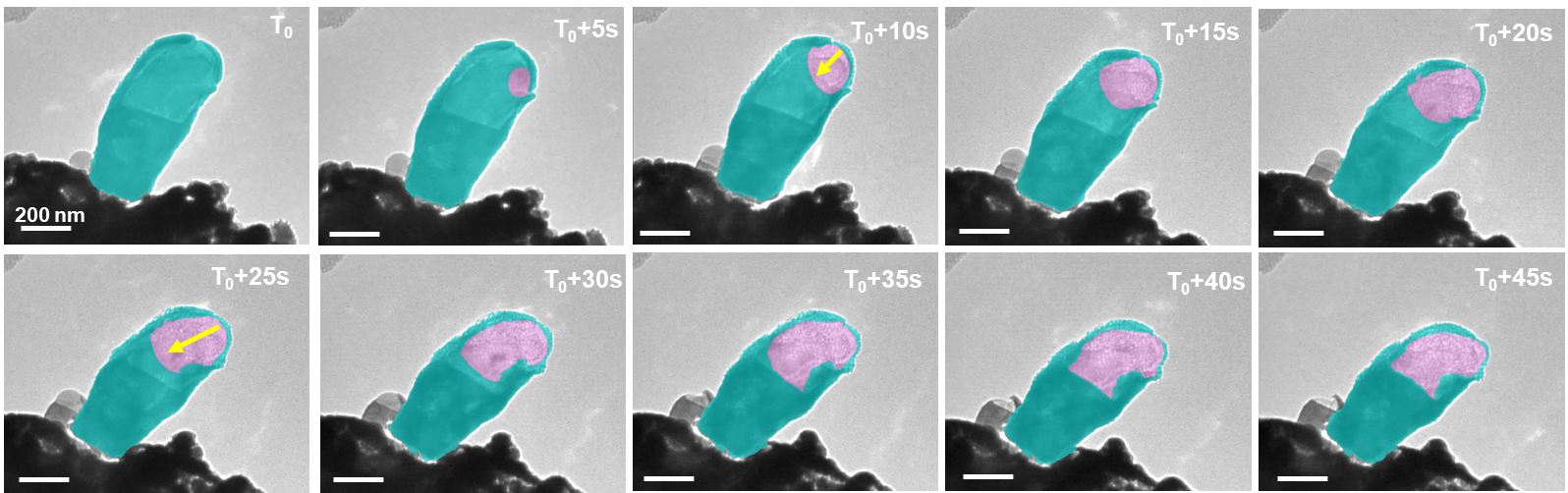


**Figure S8. Trapping the morphology change of S nanoparticle during lithiation.** The real-time image of the formation of directional cavities under TEM after mixing lithium oxide powder and sulfur powder was color-calibrated, with purple representing the cavity and blue representing the substrate, consistent with the sample in **Figure 2G**.


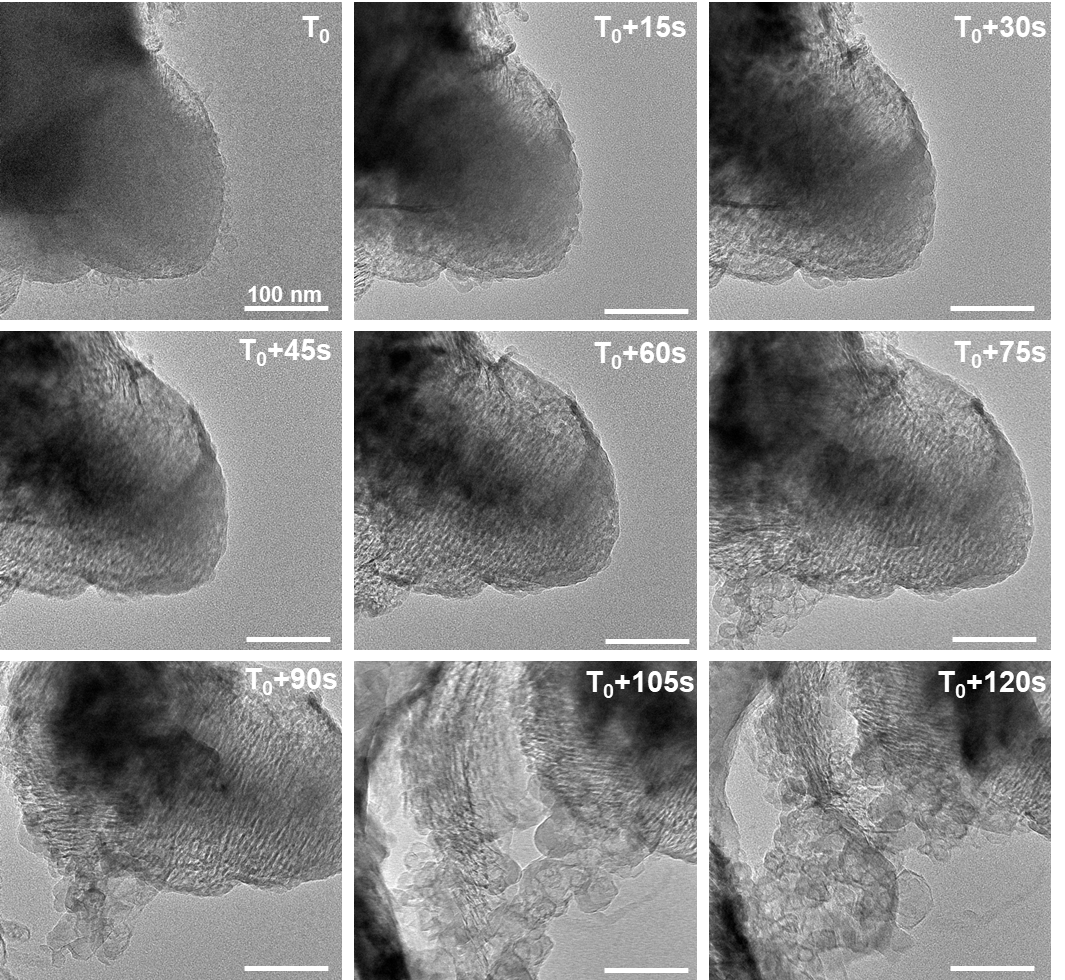


**Figure S9. Imaging the phase change and bubbles during S nanoparticle lithiation.** The snapshot image depicts the dynamic process of lithium oxide and sulfur lithiation producing a reticulated material as the HRTEM electron beam exposure time increases，consistent with the sample in **Figure 3A**.

**
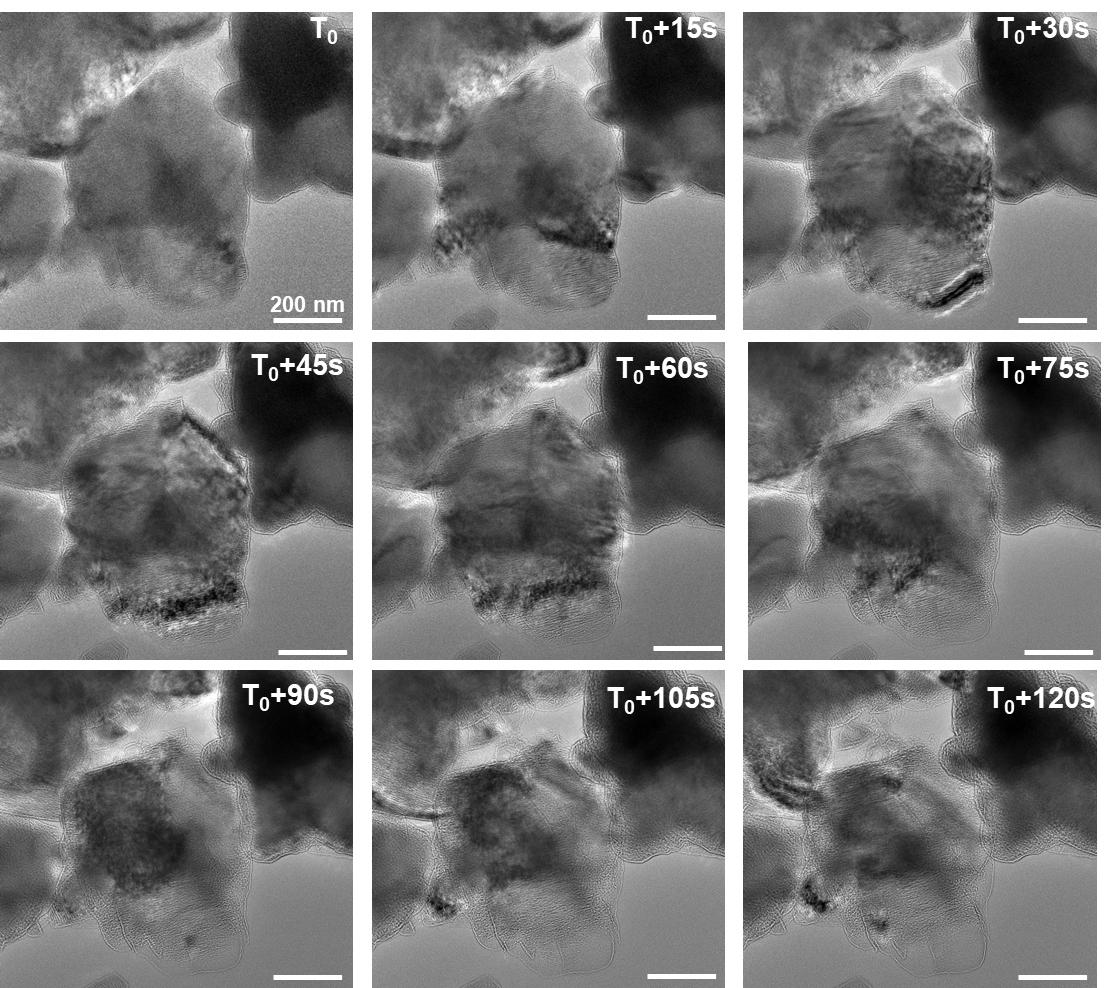
**

**Figure S10. Imaging the phase change and bubbles during S nanoparticle lithiation.** The snapshot image depicts the dynamic process of producing bubbles and reticulated matter after lithium oxide and sulfur as the exposure time of the HRTEM electron beam is prolonged, consistent with the sample in **Figure 3C**.


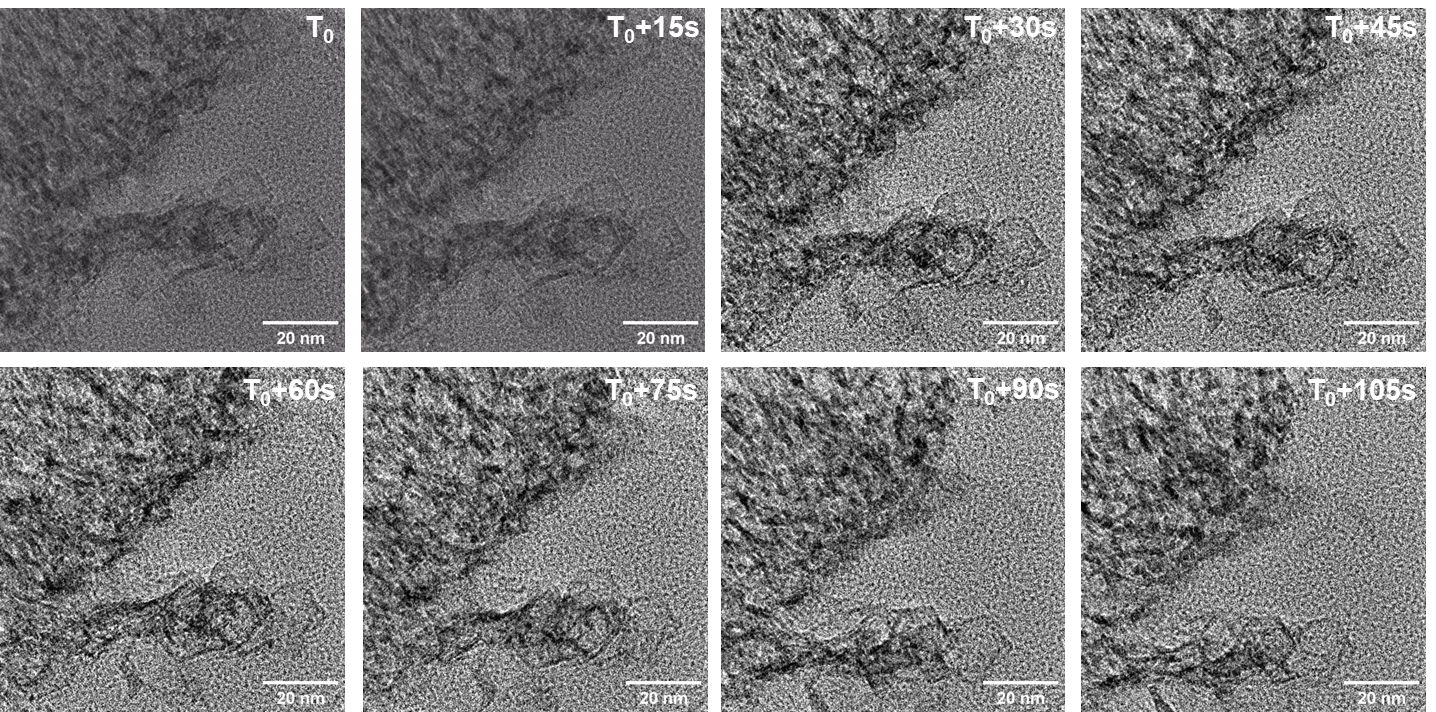


**Figure S11.** **Imaging the phase transition during sulfur lithiation.** The snapshot image depicts the phase transition process resulting from lithium oxide and sulfur lithification as the HRTEM electron beam exposure time increases, consistent with the sample in **Figure 4A**.


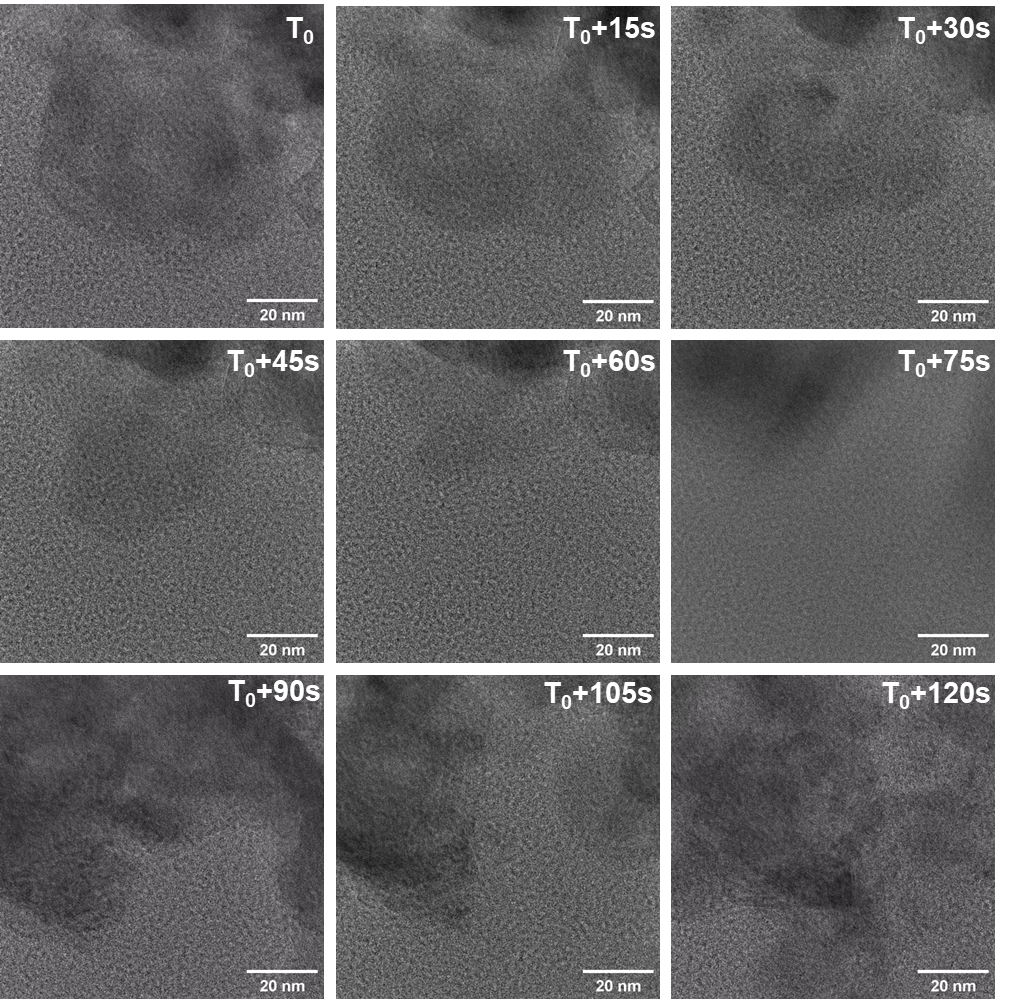


**Figure S12. Imaging the phase transition during sulfur lithiation.** The snapshot image depicts the phase transition process resulting from lithium oxide and sulfur lithification as the HRTEM electron beam exposure time increases, consistent with the sample in **Figure 4B**.


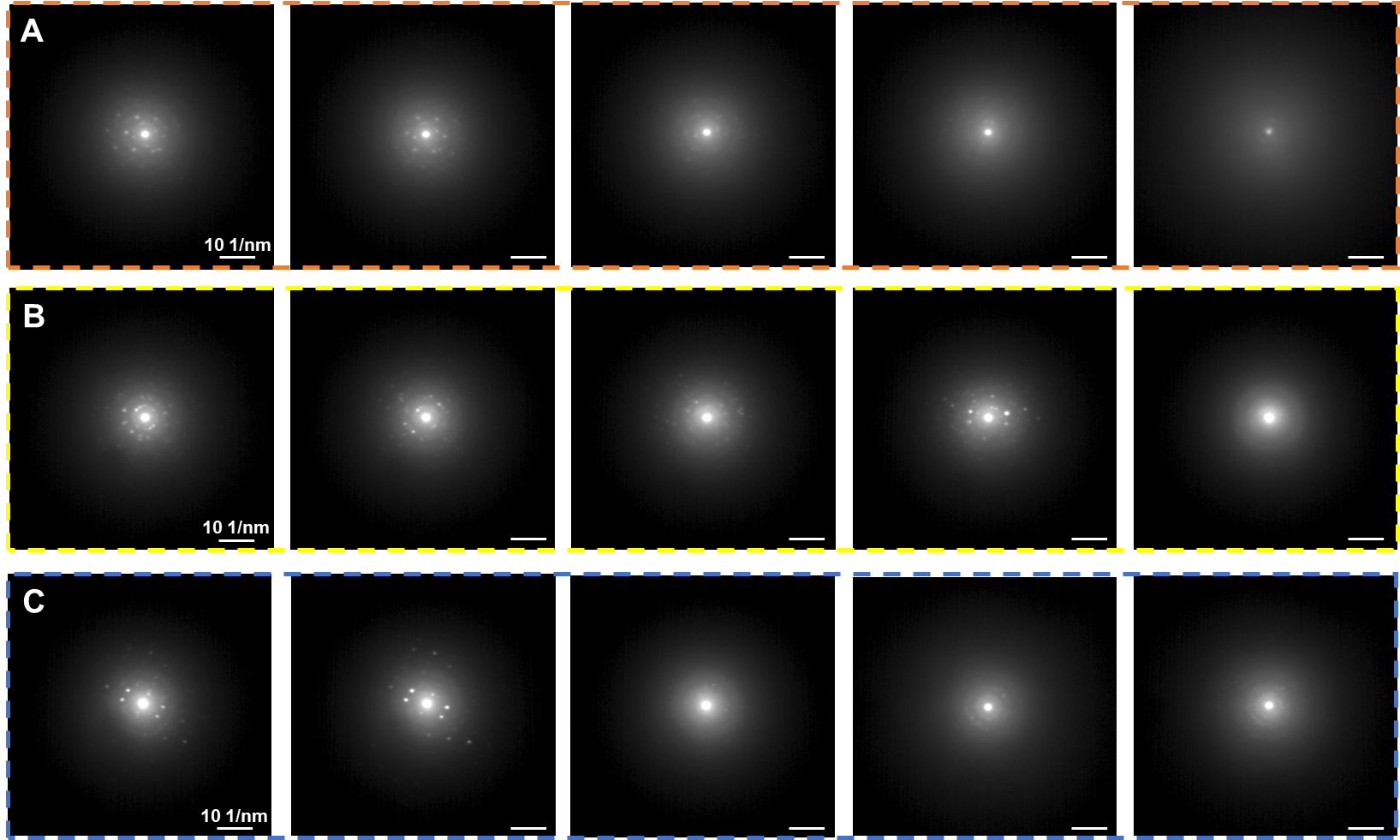


**Figure S13.** **Imaging the phase transition during S-ZIF-67-C lithiation.** After the capture of lithium oxide powder and S-ZIF-67-C powder were mixed, the diffraction results of **(A-C)** were obtained by in-situ nano diffraction test in different regions of the particle **Figure 4C.**


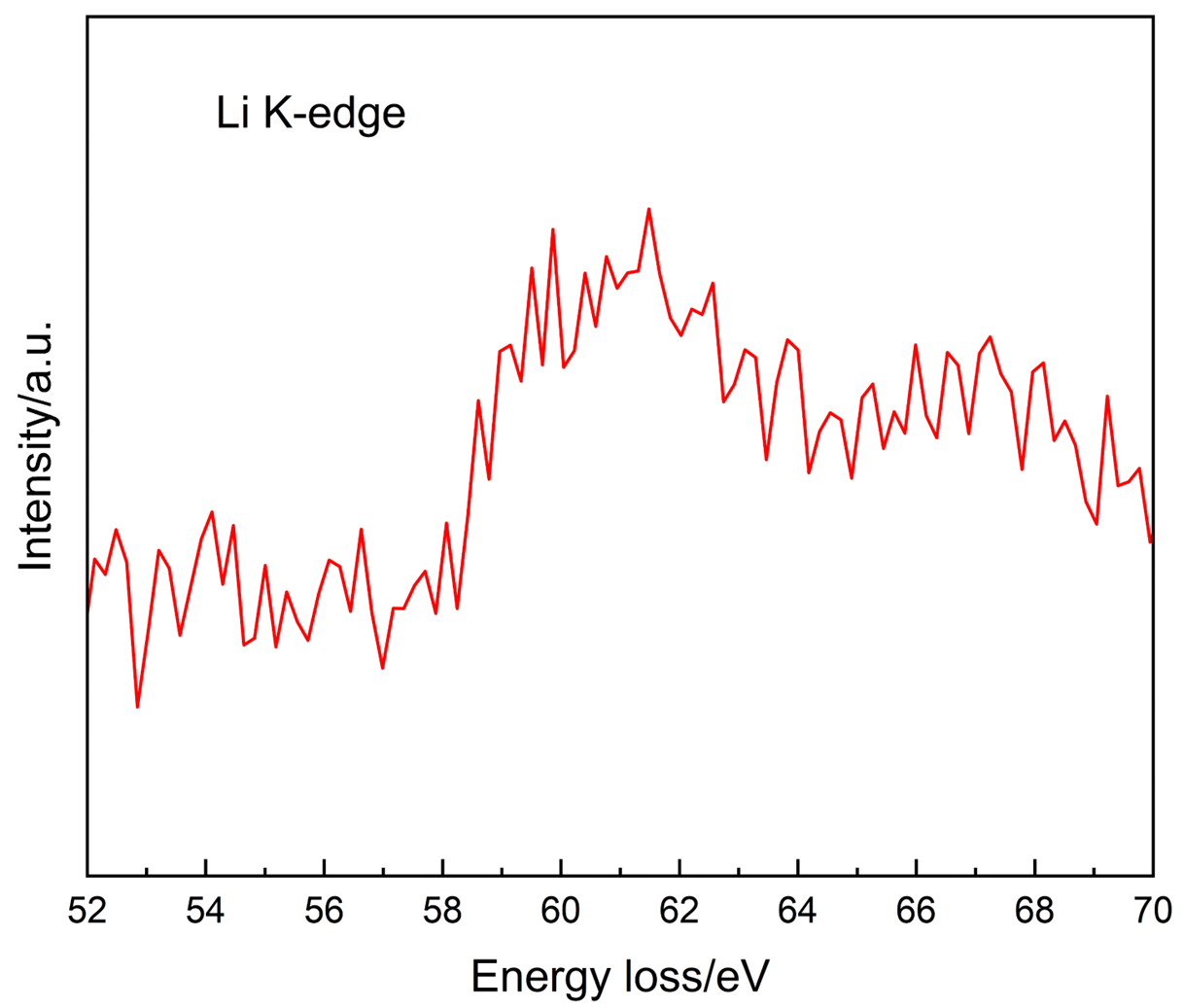


**Figure S14.** **Li-K edge of EELS spectra of S-ZIF-67-C under electron beam.** EELS detected weak Li signals within the first 3 seconds of beam irradiation.


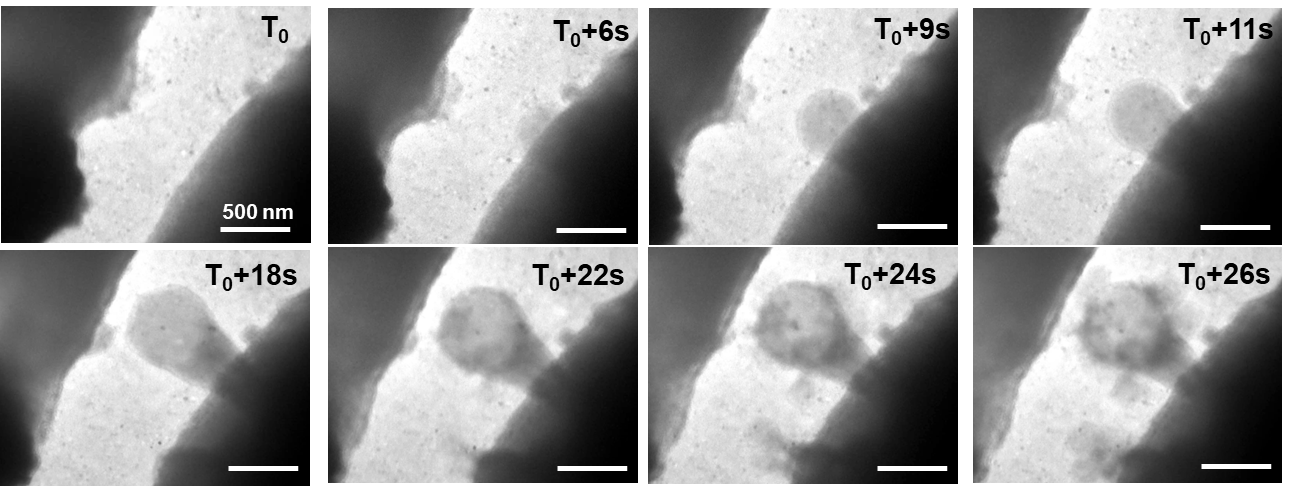


**Figure S15.** **Characterizations of polysulfide lithium created by electron beam irradiation.** The snapshot image depicts the dynamic process of lithium oxide and S-ZIF-67-C lithification as the TEM electron beam exposure time increases, consistent with the sample in **Figure 5A**.


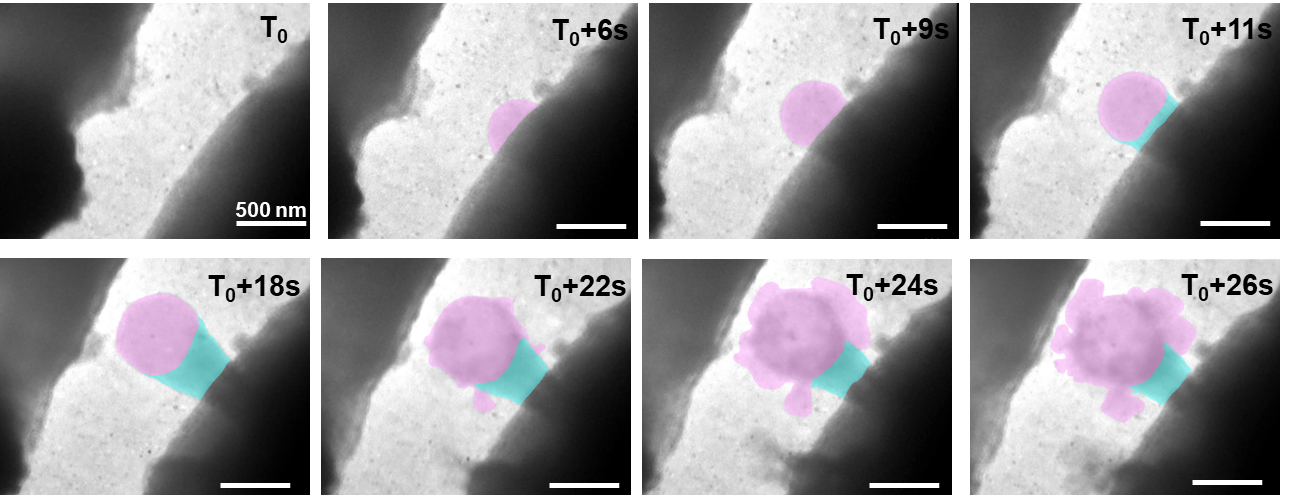


**Figure S16. Characterizations of polysulfide lithium created by electron beam irradiation.** The dynamic process is calibrated by color, purple represents spherical material, blue represents rod-like material, and the area change trend is obtained, consistent with the sample in **Figure 5B**.


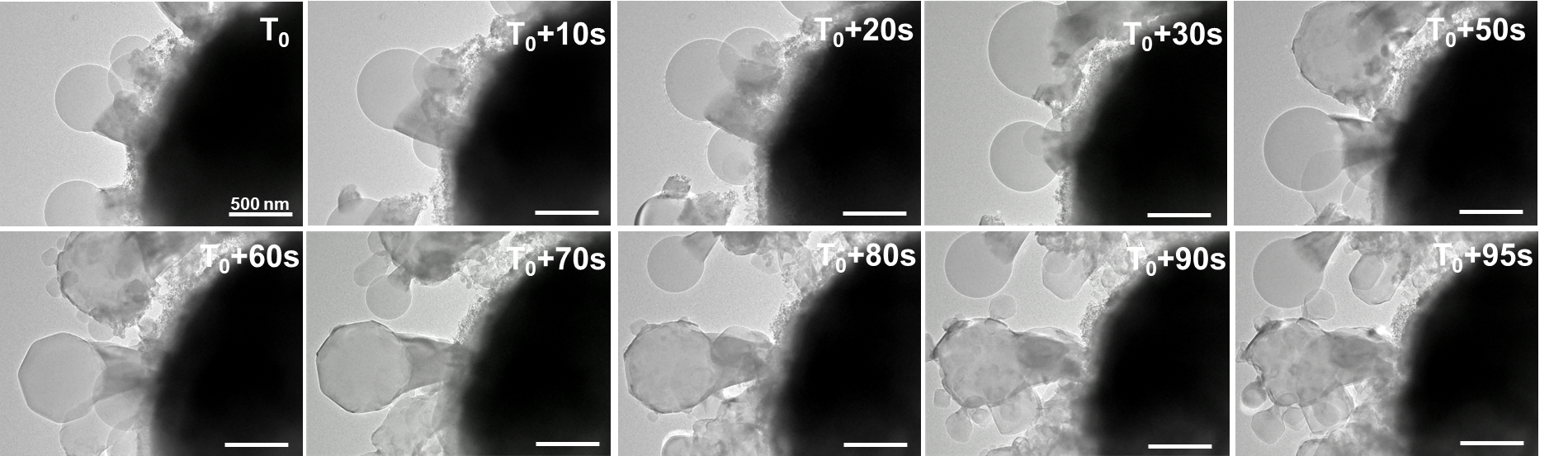


**Figure S17. Characterizations of polysulfide lithium created by electron beam irradiation.** The snapshot image depicts the dynamic process of lithium oxide and S-ZIF-67-C lithification as the TEM electron beam exposure time increases.

**
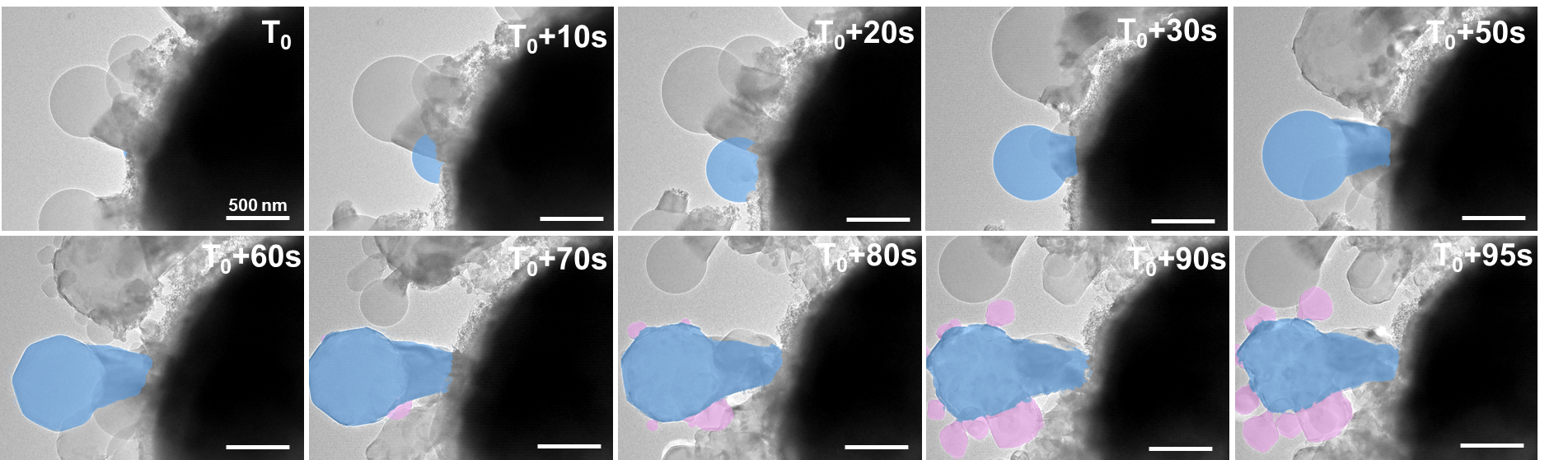
**

**Figure S18. Characterizations of polysulfide lithium created by electron beam irradiation.** Real-time images of the lithiation kinetics under TEM after mixing lithium oxide powder and sulfur-loaded toner were calibrated, with blue being the initial growth of the cue material and purple being the secondary cue material that continued to grow on top of the cue material.


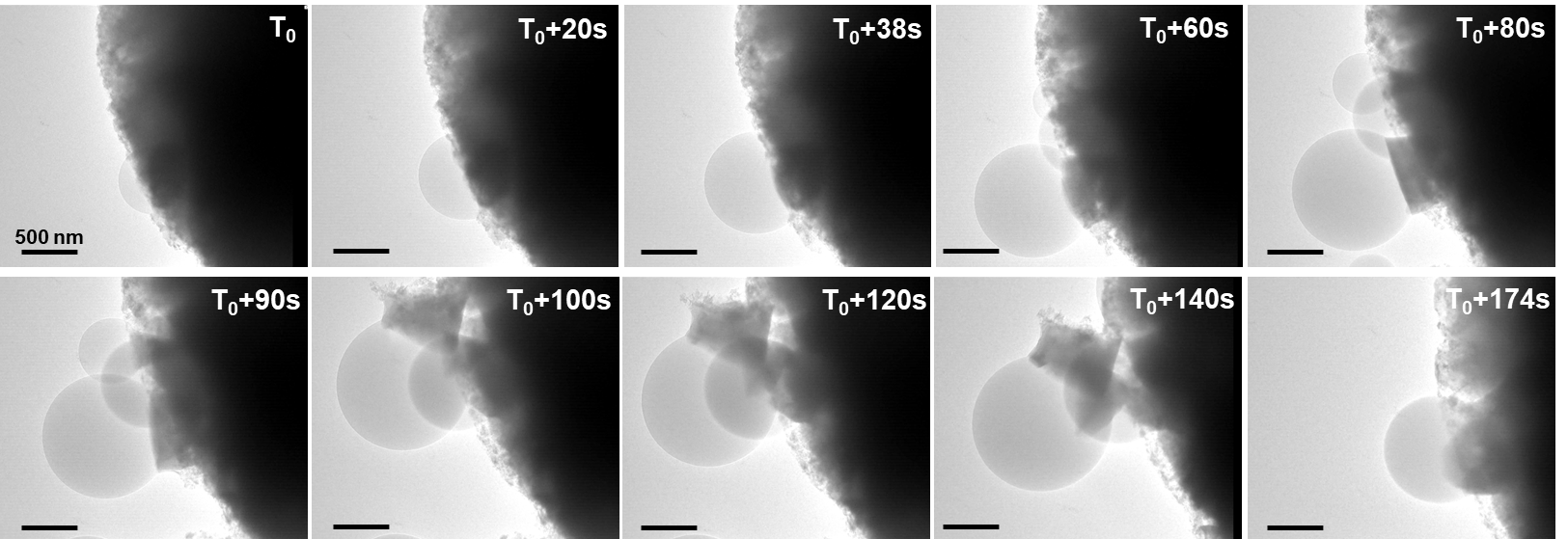


**Figure S19. Characterizations of polysulfide lithium created by electron beam irradiation.** The snapshot image depicts the dynamic process of lithium oxide and S-ZIF-67-C spherical material breaking away from the substrate as the TEM electron beam exposure time is prolonged, consistent with the sample in **Figure 5D**.


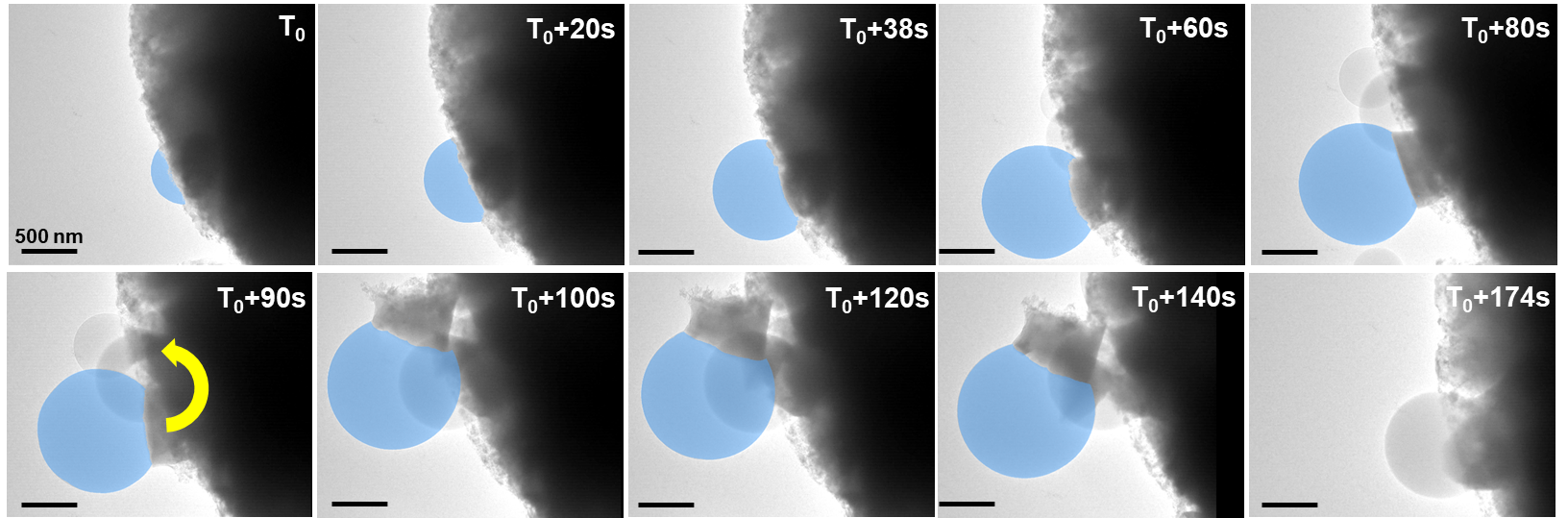


**Figure S20. Characterizations of polysulfide lithium created by electron beam irradiation.** The process of lithiation kinetics under TEM after mixing the lithium oxide powder captured in **Figure 5D** with sulfur-loading toner was calibrated using blue.


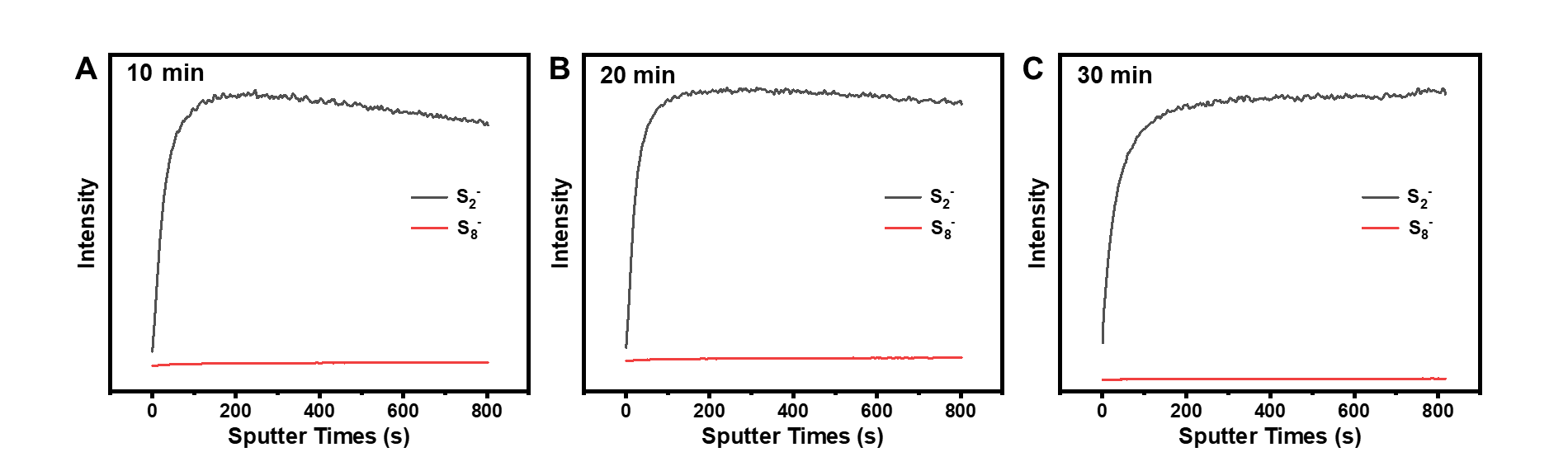


**Figure S21. TOF-SIMS depth profiling curves of the lithiated S-ZIF-67-C samples.**

**(A-C)** Representative depth profile plots of secondary ion intensities for S_2_^-^ (black curves) and S_8_^-^ (red curves) versus sputter time after exposure to the electron beam for 10 min, 20 min, and 30 min, respectively. The high intensity of the S_2_^-^ signal compared to the negligible S_8_^-^ signal across the sputtering duration indicates the effective conversion of elemental sulfur into lithiated species throughout the depth of the material. These results corroborate the 3D chemical distribution maps presented in **Figure 5F**.

**
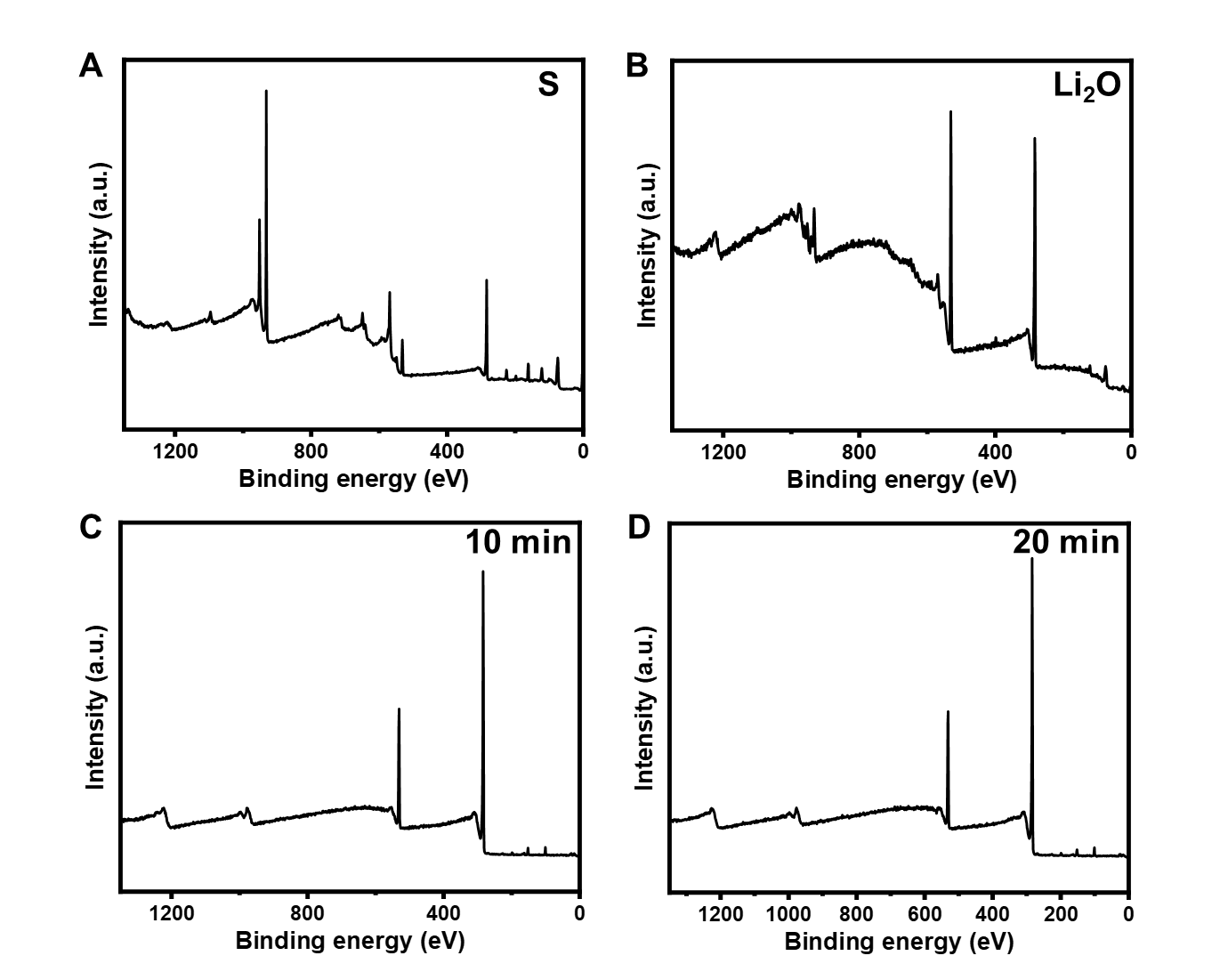
**

**Figure S22. XPS Characterizations of polysulfide lithium created by electron beam irradiation. (A)** is the XPS total spectrum of pure sulfur; **(B)** is the XPS total spectrum of pure lithium oxide; **(C)** The total spectra of XPS after mixing lithium oxide and sulfur under TEM electron beam for 10 min; **(D)** is the XPS total spectra of lithium oxide and sulfur mixed and irradiated under a TEM electron beam for 10 minutes.

**3. Supplementary Table**

**Table S1:** Detailed experimental parameters for TOF-SIMS characterization.

| **Instrument:** TOF-SIMS IONTOF M6 instrument (IONTOF GmbH) | |
| --- | --- |
| Ion species | Bi^3+^ |
| Energy | 30 keV |
| Ion current | 0.43 pA |
| Raster size | 90 µm x 90 µm |
| Mass range | 0 - 1000 u |
| Mode | High mass resolution mode |
| Sputter Ion species | Cs Ion Gun |
| Energy | 2 keV |
| Sputter area | 300 µm x 300 µm |
| Sputter rate | ~1.0 nm s^-1^ on SiO_2_ |

**Table S2**: Previous research on in-situ TEM on lithium-sulfur batteries^2, 3, 4, 5, 6, 7^

| Research observations | Journal | Publication date |
| --- | --- | --- |
| Phase Separation of Li_2_S/S at Nanoscale during Electrochemical Lithiation of the Solid‐State Lithium-Sulfur Battery Using in Situ TEM | Advanced Energy Materials | 2016 |
| In Situ TEM Observations of Discharging/Charging  of Solid-State Lithium-Sulfur Batteries at High Temperatures | Small | 2020 |
| In Situ Imaging Polysulfides Electrochemistry of Li‑S Batteries in a  Hollow Carbon Nanotubule Wet Electrochemical Cell | ACS Applied Materials & Interfaces | 2020 |
| In Situ TEM Observation of Electrochemical Lithiation of Sulfur Confined within Inner Cylindrical Pores of Carbon Nanotubes | Advanced Energy Materials | 2015 |
| Visualizing interfacial collective reaction behaviour of Li-S batteries | Nature | 2023 |
| Visualization of regulated nucleation and growth of lithium sulfides for high energy lithium sulfur batteries | Energy & Environmental Science | 2019 |

**Note:** Some of the above papers involve applying a bias voltage to simulate battery conditions and observing the lithiation process of sulfur under a TEM, while others place sulfur-carbon composites in an electrolyte containing lithium salts and use the TEM electron beam to induce the lithiation process. Listing these papers aims to highlight the innovative aspect of our work: instead of using these methods, we have mixed lithium oxide (Li_2_O) with sulfur in a solid-state configuration and used the TEM electron beam to trigger the reaction. Observing the lithiation process under these conditions reveals a different lithiation mechanism compared to previous studies, providing a reference for understanding sulfur lithiation in lithium-sulfur batteries and its characterization by electron microscopy.

**Table S3**: Previous research on in-situ TEM on lithium-sulfur batteries

| Feature | Cited Works (Nature 2023, EES 2019)^6, 7^ | This Work |
| --- | --- | --- |
| Driving Force | Electrochemical Potential (Bias/Voltage) | Electron Beam Irradiation (Radiolysis & Thermal) |
| Environment | Liquid electrolyte or simulated cell | Solid-state vacuum (Li_2_O + S powder) |
| Key Observation | Regulated nucleation, interfacial collective behavior | Explosive expansion (8300%), Directional etching |
| Scientific Goal | Optimize battery electrochemical performance | Reveal beam-induced mechanisms & artifacts |

**Table S4:** Electron beam dose accumulation for key experimental observations

| Figure / Movie | Observed Phenomenon | Electron Dose Rate (e Å ^−2^⋅s^−1^) | Duration / Timestamp (s) | Accumulated Dose (e Å ^−2^) |
| --- | --- | --- | --- | --- |
| Fig. 1B / Movie S1 | Volatilization of pure Sulfur | 12.2 | 18 | ~220 |
| Fig. 1D / Movie S2 | Shrinkage of pure Li_2_O | 12.2 | 168 | ~2050 |
| Fig. 2A / Movie S3 | Explosive Lithiation of S | 12.2 | 34 | ~415 |
| Fig. 2D / Movie S6 | Shrinkage of mixed powder | 12.2 | 88 | ~1074 |
| Fig. 2F / Movie S7 | Directional Cavity Formation | 12.2 | 45 | ~549 |
| Fig. 3A / Movie S8 | Formation of Reticulated Material (HRTEM) | 14.5 | 120 | ~1740 |
| Fig. 4A / Movie S10 | Phase Transition (Li_2_O_2_ formation) | 14.5 | 106 | ~1537 |
| Fig. 4B / Movie S11 | Phase Transition (Li_2_S formation) | 14.5 | 120 | ~1740 |
| Fig. 5A / Movie S15 | Lithiation of S-ZIF-67-C | 12.2 | 26 | ~317 |
| Fig. 5D / Movie S17 | Shedding of product from ZIF-67-C | 12.2 | 174 | ~2123 |
| Fig. 6A / Movie S18 | Cryo-TEM at -50 °C (Stable state) | 35 | 508 | ~152400 |
| Fig. 6B / Movie S19 | Cryo-TEM at -150 °C (Stable state) | 35 | 160 | ~5600 |

**Table S5:** Electron beam damage threshold study: effect of dose rate and temperature

| Dose Rate (e Å ^-2^ s^-1^) | Temperature | Observation (Kinetic Response) | Criticality Assessment | Source |
| --- | --- | --- | --- | --- |
| 10.0 | 25 °C | Slow Phase Transition: Transformation from crystalline to amorphous; no explosive expansion. | Near Activation Energy: Sufficient to drive slow phase evolution but enables capture of intermediate states. | Fig. 4C / Movie S12 |
| 12.2 | 25 °C | Explosive Lithiation: Rapid volume expansion (8300%) and violent S-Li reaction. | Thermal Runaway: Energy input exceeds critical threshold, driving rapid, thermally-dominated kinetics. | Fig. 2A / Movie S3 |
| 12.2 | -50 °C | Stable State: No significant morphological changes observed. | Below Threshold: Thermal energy accumulation is insufficient to activate the reaction at this flux. | Fig. 6A / Movie S18 |
| 35.0 | -50 °C | Beam-Induced Damage: Shrinkage of Li_2_O and initiation of reaction. | Critical Threshold Reached: High dose rate overcomes the cryogenic stabilization at -50 °C. | Fig. 6A (Text) / Movie S18 |
| 12.2 – 35.0 | -150 °C | Complete Suppression: Target particles remain stable with diameter fluctuations < ±2 nm. | Fully Suppressed: Ultra-low temperature effectively dissipates beam-induced heat, preventing reaction even at high doses. | Fig. 6B / Movie S19 |

**4. Supplementary Movies S1-S20**

**Movie S1:** Synchronized in-situ TEM movie showing “A dynamic process in which pure sulfur powder gradually volatilizes and disappears from the liquid state with the extension of TEM electron beam irradiation time”. The movie corresponds to the text **Figure 1B**.

Electron dose rate: 12.2 e Å^-2^ s^-1^; TEM model: JEM-1400FLASH; Accelerating voltage: 120 kV; Playback rate: real speed.

**Movie S2:** Synchronized in-situ TEM movie showing “A dynamic process in which lithium oxide powder gradually shrinks as the TEM electron beam exposure time increases”. The movie corresponds to the text **Figure 1D**.

Electron dose rate: 12.2 e Å^-2^ s^-1^; TEM model: JEM-1400FLASH; Accelerating voltage: 120 kV; Playback rate: 25 times x real speed.

**Movie S3:** Synchronized in-situ TEM movie showing “With the extended exposure time of the TEM electron beam, the dynamic process of explosive lithiation of lithium oxide and sulfur powder”. The movie corresponds to the text **Figure 2A** and **2B**.

Electron dose rate: 12.2 e Å^-2^ s^-1^; TEM model: JEM-1400FLASH; Accelerating voltage: 120 kV; Playback rate: 5 x real speed.

**Movie S4:** Synchronized in-situ TEM movie showing “A replicate dynamic process in which lithium oxide powder gradually shrinks as the TEM electron beam exposure time increases”. This movie serves as an additional observation to confirm the reproducibility of the phenomenon shown in Movie S2 and corresponds to the text **Figure S4.**

Electron dose rate: 12.2 e Å^-2^ s^-1^; TEM model: JEM-1400FLASH; Accelerating voltage: 120 kV.

**Movie S5:** Synchronized in-situ TEM movie showing “The dynamic behavior of aluminum oxide (Al_2_O_3_) or magnesium oxide (MgO) and sulfur mixed powder under electron beam irradiation”. This experiment serves as a control to verify the mechanism of the explosive expansion. Unlike the Li_2_O-S system, no significant volume expansion or explosive reaction was observed in this non-lithium system under identical irradiation conditions, confirming that the "explosive" phenomenon reported in **Figure 2** is driven by the lithiation reaction initiated by Li_2_O decomposition and corresponds to the text **Figure S5**.

Electron dose rate: 12.2 e Å^-2^ s^-1^; TEM model: JEM-1400FLASH; Accelerating voltage: 120 kV; Playback rate: 30 x real speed.

**Movie S6:** Synchronized in-situ TEM movie showing “A dynamic process in which the lithium oxide and sulfur mixed powder shrinks and disappears as the TEM electron beam exposure time increases”. The movie corresponds to the text **Figure 2D**.

Electron dose rate: 12.2 e Å^-2^ s^-1^; TEM model: JEM-1400FLASH; Accelerating voltage: 120 kV; Playback rate: 25 x real speed.

**Movie S7:** Synchronized in-situ TEM movie showing “As the TEM electron beam exposure time increases, the dynamic process of lithium oxide and sulfur powder forming directional cavities”. The movie corresponds to the text **Figure 2F** and **2G**.

Electron dose rate: 12.2 e Å^-2^ s^-1^; TEM model: JEM-1400FLASH; Accelerating voltage: 120 kV; Playback rate: 25 x real speed.

**Movie S8:** Synchronized in-situ TEM movie showing “As the HRTEM electron beam exposure time increases, lithium oxide and lithium sulfur lithiation produce a dynamic process of mesh material”. The movie corresponds to the text **Figure 3A**.

Electron dose rate: 14.5 e Å^-2^ s^-1^; TEM model: Talos F200X G2; Accelerating voltage: 200 kV; Playback rate: 30 x real speed.

**Movie S9:** Synchronized in-situ TEM movie showing “With the increase in HRTEM electron beam exposure time, lithium oxide and sulfur are followed by a dynamic process of bubbles and reticulated substances”. The movie corresponds to the text **Figure 3C**.

Electron dose rate: 14.5 e Å^-2^ s^-1^; TEM model: Talos F200X G2; Accelerating voltage: 200 kV; Playback rate: 30 x real speed.

**Movie S10:** Synchronized in-situ TEM movie showing “With the increase of HRTEM electron beam exposure time, the phase transition process produced by lithium oxide and lithium sulfur petrification”. The movie corresponds to the text **Figure 4A**.

Electron dose rate: 14.5 e Å^-2^ s^-1^; TEM model: Talos F200X G2; Accelerating voltage: 200 kV; Playback rate: 30 x real speed.

**Movie S11:** Synchronized in-situ TEM movie showing “With the increase of HRTEM electron beam exposure time, the phase transition process produced by lithium oxide and lithium sulfur petrification”. The movie corresponds to the text **Figure 4B**.

Electron dose rate: 14.5 e Å^-2^ s^-1^; TEM model: Talos F200X G2; Accelerating voltage: 200 kV; Playback rate: 30 x real speed.

**Movie S12:** In-situ nano diffraction test with the orange box in **Figure 4C.**

Electron dose rate: 10 e Å^-2^ s^-1^; TEM model: Talos F200X G2; Accelerating voltage: 200 kV; Playback rate: 1 x real speed.

**Movie S1****3:** In-situ nano diffraction test with the yellow box in **Figure 4C**.

Electron dose rate: 10 e Å^-2^ s^-1^; TEM model: Talos F200X G2; Accelerating voltage: 200 kV; Playback rate: 1 x real speed.

**Movie S1****4:** In-situ nano diffraction test with the blue box in **Figure 4C**.

Electron dose rate: 10 e Å^-2^ s^-1^; TEM model: Talos F200X G2; Accelerating voltage: 200 kV; Playback rate: 1 x real speed.

**Movie S15:** Synchronized in-situ TEM movie showing “Dynamic processes of lithium oxide and S-ZIF-67-C lithiation as the TEM electron beam exposure time increased”. The movie corresponds to the text **Figure 5A** and **5B**.

Electron dose rate: 12.2 e Å^-2^ s^-1^; TEM model: JEM-1400FLASH; Accelerating voltage: 120 kV; Playback rate: 5 x real speed.

**Movie S16:** Synchronized in-situ TEM movie showing “Dynamic processes of lithium oxide and S-ZIF-67-C lithiation as the TEM electron beam exposure time increased”. The movie corresponds to the text **Figures S17** and **S18**.

Electron dose rate: 12.2 e Å^-2^ s^-1^; TEM model: JEM-1400FLASH; Accelerating voltage: 120 kV; Playback rate: 25 x real speed.

**Movie S17:** Synchronized in-situ TEM movie showing “The dynamic process of lithium oxide and S-ZIF-67-C spherical material shedding from the substrate as the TEM electron beam exposure time increases”. The movie corresponds to the text **Figure 5D**.

Electron dose rate: 12.2 e Å^-2^ s^-1^; TEM model: JEM-1400FLASH; Accelerating voltage: 120 kV; Playback rate: 30 x real speed.

**Movie S18:** Synchronized in-situ TEM movie showing “The dynamic process of TEM imaging of lithium oxide and sulfur mixed powders at -50 °C”. The movie corresponds to the text **Figure 6A**.

Electron dose rate: 12.2 e Å^-2^ s^-1^, 35 e Å^-2^ s^-1^; TEM model: Talos F200C; Accelerating voltage: 200 kV; Temperature: -50 °C; Playback rate: 25 x real speed.

**Movie S1****9:** Synchronized in-situ TEM movie showing “The dynamic process of TEM imaging of lithium oxide and sulfur mixed powders at -150 °C”. The movie corresponds to the text **Figure 6B**.

Electron dose rate: 12.2 e Å^-2^ s^-1^, 35 e Å^-2^ s^-1^; TEM model: Talos F200C; Accelerating voltage: 200 kV; Temperature: -150 °C; Playback rate: 25 x real speed.

**Movie S20: MD** Simulated movie showing the kinetics of sulfur lithiation under electron beam irradiation.

**References**

1. Sun Y*, et al.* Revealing microscopic dynamics: in situ liquid-phase TEM for live observations of soft materials and quantitative analysis via deep learning. *Nanoscale* **16**, 2945-2954 (2024).

2. Yang Z*, et al.* Phase Separation of Li 2 S/S at Nanoscale during Electrochemical Lithiation of the Solid-State Lithium-Sulfur Battery Using In Situ TEM. *Adv. Energy Mater.* **6**, 1600806 (2016).

3. Wang Z, Tang Y, Zhang L, Li M, Shan Z, Huang J. In situ TEM observations of discharging/charging of solid‐state lithium‐sulfur batteries at high temperatures. *Small* **16**, 2001899 (2020).

4. Wang Z*, et al.* In situ imaging polysulfides electrochemistry of Li-S batteries in a hollow carbon nanotubule wet electrochemical cell. *ACS Appl. Mater. Interfaces* **12**, 55971-55981 (2020).

5. Kim H, Lee JT, Magasinski A, Zhao K, Liu Y, Yushin G. In situ TEM observation of electrochemical lithiation of sulfur confined within inner cylindrical pores of carbon nanotubes. *Adv. Energy Mater.* **5**, 1501306 (2015).

6. Zhou S*, et al.* Visualizing interfacial collective reaction behaviour of Li-S batteries. *Nature* **621**, 75-81 (2023).

7. Xu Z-L*, et al.* Visualization of regulated nucleation and growth of lithium sulfides for high energy lithium sulfur batteries. *Energy Environ. Sci.* **12**, 3144-3155 (2019).
